# Targeted Micellar Phthalocyanine for Lymph Node Metastasis Homing and Photothermal Therapy in an Orthotopic Colorectal Tumor Model

**DOI:** 10.1007/s40820-021-00666-8

**Published:** 2021-06-19

**Authors:** Hai-Yi Feng, Yihang Yuan, Yunpeng Zhang, Hai-Jun Liu, Xiao Dong, Si-Cong Yang, Xue-Liang Liu, Xing Lai, Mao-Hua Zhu, Jue Wang, Qin Lu, Quanjun Lin, Hong-Zhuan Chen, Jonathan F. Lovell, Peng Sun, Chao Fang

**Affiliations:** 1 Hongqiao International Institute of Medicine, Tongren Hospital and State Key Laboratory of Oncogenes and Related Genes, Department of Pharmacology and Chemical Biology, Shanghai Jiao Tong University School of Medicine (SJTU-SM), Shanghai, 200025 People’s Republic of China; 2 Department of General Surgery, Tongren Hospital, SJTU-SM, Shanghai, 200336 People’s Republic of China; 3 Institute of Interdisciplinary Integrative Biomedical Research, Shuguang Hospital, Shanghai University of Traditional Chinese Medicine, Shanghai, 201203 People’s Republic of China; 4 Department of Biomedical Engineering, University at Buffalo, State University of New York, Buffalo, NY 14260 USA

**Keywords:** Lymph node metastasis, Photothermal therapy, Trastuzumab, Phthalocyanine, Micelles

## Abstract

- Small-sized trastuzumab-targeted micelles (T-MP) were engineered using a surfactant-stripping approach that yielded concentrated phthalocyanines with strong near infrared absorption.
- T-MP accumulated more in the lymph node (LN) metastases of orthotopic colorectal cancer compared to the micelles conjugated with control IgG.
- Following surgical resection of the primary tumor, minimally invasive photothermal treatment of the metastatic LN with T-MP, but not the control micelles, extended mouse survival.

**Abstract:**

Tumor lymph node (LN) metastasis seriously affects the treatment prognosis. Studies have shown that nanoparticles with size of sub-50 nm can directly penetrate into LN metastases after intravenous administration. Here, we speculate through introducing targeting capacity, the nanoparticle accumulation in LN metastases would be further enhanced for improved local treatment such as photothermal therapy. Trastuzumab-targeted micelles (< 50 nm) were formulated using a unique surfactant-stripping approach that yielded concentrated phthalocyanines with strong near-infrared absorption. Targeted micellar phthalocyanine (T-MP) was an effective photothermal transducer and ablated HT-29 cells in vitro. A HER2-expressing colorectal cancer cell line (HT-29) was used to establish an orthotopic mouse model that developed metastatic disease in mesenteric sentinel LN. T-MP accumulated more in the LN metastases compared to the micelles conjugated with control IgG. Following surgical resection of the primary tumor, minimally invasive photothermal treatment of the metastatic LN with T-MP, but not the control micelles, extended mouse survival. Our findings demonstrate for the first time that targeted small-sized nanoparticles have potential to enable superior paradigms for dealing with LN metastases.
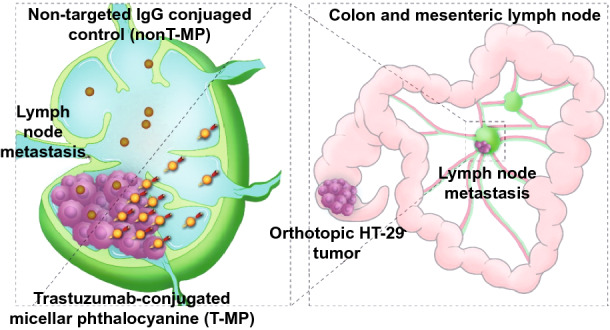

**Supplementary Information:**

The online version contains supplementary material available at 10.1007/s40820-021-00666-8.

## Introduction

Solid tumor progression is usually accompanied by lymphatic metastasis, which seriously affects the disease prognosis [1, 2]. Lymphadenectomy is the dominant method for treating lymphatic metastasis in cancer clinic [3]. However, this surgical operation is mainly based on surgeons’ clinical practice experience, such as the D2 lymphadenectomy for gastric cancer management [4], which does not accurately distinguish between metastatic and healthy lymph nodes (LNs). Due to the lack of reliable method for metastasis identification and lymphatic imaging, precise clearance of the metastatic LNs still remains a big challenge. Incomplete lymphadenectomy may finally end up with disease recurrence, and excessive resection would lead to a higher incidence of postoperative complications [3, 4]. Near-infrared (NIR) fluorescent imaging after peritumoral injection of indocyanine green (ICG) offers an effective method to visualize the lymphatic anatomy for surgeons [5]. However, the local injection of the imaging agent is restricted due to the disadvantage of “dye spillage” which makes high background signals [6].

Systemic chemotherapy for the LN metastasis usually requires high doses and inevitably increases the risk of dose-limiting toxicities [7]. Thus, eradicating the tumor cells in the LN metastases by targeted drug delivery to the metastatic LNs is a more attractive solution [8]. However, most of the preclinical efforts on tumor metastatic LN targeted drug delivery utilize the subcutaneous tumor model, such as the mostly used xenograft model in the mouse footpad [9, 10]. Nanoparticles are administered through the interstitial space next to the tumor to target locally metastatic LNs. Generally, such tumor models and local delivery methods are lack of clinical relevance. In fact, in contrast to lots of reports aiming to improve nanoparticle penetration in tumor primary site [11], relatively few efforts have been made for LN metastasis penetration and distribution.

Recently, systemic administration of nanoparticles has been explored to target LN metastasis. Through enhancing the penetration in tumor tissues by pH-responsive size reduction from 100 to 5 nm at the tumor site, nanoparticles were allowed to enter into the LNs through tumor-draining lymphatics [6, 12]. Lymphatic vessels of solid tumors are poorly developed, which is a key pathophysiological basis of the enhanced permeation and retention (EPR) effect [13–15], and in most clinical cancer settings, chemotherapy is performed after the primary tumor is resected. Therefore, the application of this method after tumor resection might be restricted. Compared with large-sized particle, intravenously administered nanocarriers with smaller dimension (< 50 nm) can specifically extravasate from the blood vessels in LN metastases and accumulate, while their distribution in healthy LNs was significantly low [7]. This size-controlled targeting to the LN metastasis does not rely on the existence of the primary tumor [7] and thus holds potential for wider application.

It has been shown that tumor cell-targeted nanocarriers can accumulate more in the primary lesion of solid tumors compared the non-targeted counterparts and therefore confer enhanced therapeutic outcome [16]. We thus hypothesize that through endowing nanocarriers with targeting property for tumor cells, furtherly enhanced nanoparticle accumulation in LN metastasis would be achieved, which may provide greater benefits for the local treatment (such as photothermal therapy) [17, 18] of LN metastasis.

**Scheme 1:**
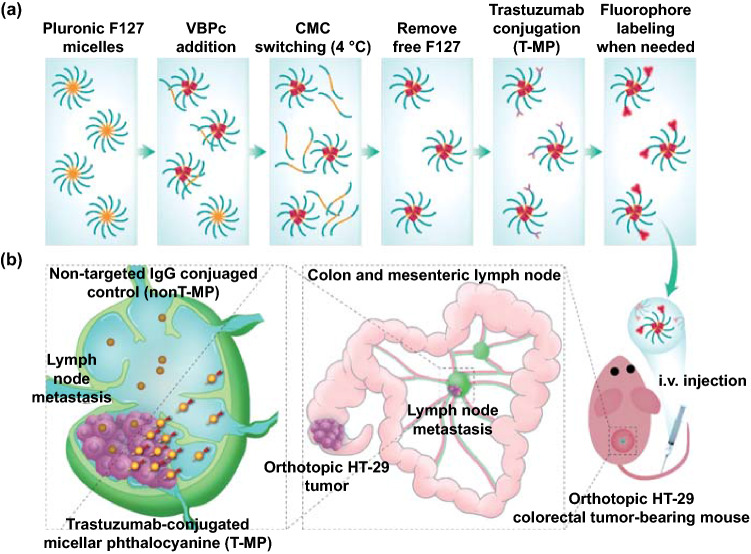
Schematic illustration of the engineering of surfactant-stripped targeted micellar phthalocyanine (T-MP) (**a**) and improved LN metastasis targeting using this approach (**b**)

HER2 (human epidermal growth factor receptor 2)-targeted micellular phthalocyanine with size below 50 nm was developed as illustrated in Scheme 1a. The near-infrared (NIR) chromophore VBPc (Vanadyl 3,10,17,24-tetra-tert-butyl-1,8,15,22-tetrakis(dimethylamino)-29H,31H-phthalocyanine, a photothermal and photoacoustic inducer) was loaded into carboxylated F127 micelles using the emerging surfactant-stripping strategy, taking advantage of the temperature-dependent CMC (critical micelle concentration) of F127 that enables removal of free and loosely bound surfactant using membrane filtration at 4 °C [19–21]. This generates concentrated VBPc micelles containing minimal solubilizing excipient and intense NIR absorption to enable both photoacoustic imaging and photothermal properties. Next, trastuzumab, a human anti-HER2 antibody, was conjugated to the micelle surface through EDS/NHS chemistry to generate trastuzumab-conjugated targeted micellar phthalocyanine (T-MP). T-MP would enable targeting to HER2 expressed on the surface of tumor cells, such as HT-29 colorectal cancer cells [22, 23]. An orthotopic HT-29 colorectal tumor model was established [24], and metastasis to the mesenteric sentinel LN was observed. It was postulated that after intravenous (i.v.) injection, the small-sized T-MP can accumulate more in the LN metastasis compared to the non-targeted isotype IgG-conjugated controls (nonT-MP) (Scheme 1b), which may lead to an improved photothermal therapy for the LN metastases. To our knowledge, this work is the first report demonstrating that through targeting modification, i.v. injected small-sized nanoparticles can achieve more accumulation in LN metastases for improved therapeutic outcomes (photothermal treatment in this study), which are also superior to the traditional lymphadenectomy that usually incurred surgical trauma and postoperative complications. The multifunctional properties of T-MP were characterized, and their potency in overcoming LN metastasis in the metastatic orthotopic HT-29 colorectal tumor model was investigated.

## Experimental Section

### Materials, Cell Culture, and Animals

Pluronic F127 and Vanadyl 3,10,17,24-tetra-tert-butyl-1,8,15,22-tetrakis(dimethylamino)-29H,31H-phthalocyanine (VBPc) were supplied by Sigma-Aldrich (Shanghai, China). 1-ethyl-3-(3-dimethylaminopropyl) carbodiimide (EDC) and N-hydroxysuccinimide (NHS) were obtained from Aladdin Chemistry Company (Shanghai, China). Trastuzumab (Herceptin) was obtained from Tongren Hospital (Shanghai, China). Rabbit anti-HER2 antibody and FITC/HRP goat anti-rabbit antibody were obtained from Abcam (Shanghai, China). Cy5.5 NHS ester was purchased from Lumiprobe (Hunt Valley, Maryland). iFluor 594 NHS ester was obtained from AAT Bioquest (Sunnyvale, CA). McCoy’s 5A Medium, fetal bovine serum (FBS), Dulbecco's phosphate-buffered saline (DPBS), penicillin, and streptomycin were purchased from Thermo Fisher Scientific. Double distilled water was purified using a Millipore simplicity system (Millipore, Bedford, MA). All other chemicals were of analytical grade and used without further purification.

HT-29 human colorectal adenocarcinoma cell line was obtained from the ATCC (Manassas, VA). HT-29 cells transfected with luciferase (HT-29-luc) were constructed by Shanghai Model Organisms Center (Shanghai, China). Cells were cultured in McCoy’s 5A medium with 10% FBS, 10^5^ U L^−1^ penicillin, and 100 mg L^−1^ streptomycin.

Female BALB/c nude mice (4–5 weeks old) were provided by Shanghai Laboratory Animal Center (Chinese Academy of Sciences, Shanghai, China). The animal experiment designed in this study was approved by the ethical committee of SJTU-SM.

### Preparation and Characterization of Targeted Micellar Phthalocyanine (T-MP)

The micellular phthalocyanine was formed through self-assembly of Pluronic F127 and VBPc [19]. 2 mg VBPc in 1 mL dichloromethane was added dropwise to 10 mL 10% (w/v) Pluronic F127 (containing 15% carboxylated polymer prepared as described) [25]. The solution was stirred at room temperature until the dichloromethane was evaporated. After centrifugation at 5000*g* for 5 min, the supernatant was purified through CMC switching. For this procedure, ultrafiltration using Amicon Ultra-15 centrifugal filtration device (100 KD MWCO) at 4 °C was performed until 200 μL solution was retained. Distilled water was then added back into the filtration device, and the washing procedure was repeated twice.

Antibody modification is a common route to confer targeting property of the nanocarriers [26]. For trastuzumab conjugation, the antibody was mixed with the micellar VPBc at 1:3 molar ratio of trastuzumab to carboxyl groups of micelles in the presence of EDC and NHS. The solution was then stirred for 12 h at 4 °C. The resulting targeted micellar phthalocyanine (T-MP) was purified by ultrafiltration to remove excess EDC and NHS.

The VBPc fluorescence was quenched due to the aggregation in the micellar core. For the fluorescence labeling of T-MP, Cy5.5 or iFluor 594 NHS ester was incubated with T-MP in dark for 1 h (1:5 molar ratio of trastuzumab to dye). Excess dyes were removed by ultrafiltration at 5000*g* for 3 times.

The morphology of the resulting T-MP was observed using transmission electron microscopy (TEM, FEI Talos F200X). The hydrodynamic size and zeta potential of nanoparticles were determined through dynamic light scattering (DLS) method and measured by ZetaSizer Nano ZS instrument (Malvern, Worcestershire, the UK). Vis–NIR absorbance of free VBPc in dichloromethane and unmodified MP in water was examined on a spectrofluorophotometer (Thermo Scientific Varioskan Flash). The carboxylation of F127 was examined using Fourier transform infrared spectroscopy (FTIR). The antibody conjugation was identified using FTIR spectroscopy and X-ray photoelectron spectroscopy (XPS) assay. The conjugated trastuzumab and the linked Cy5.5 fluorophore on the micelles were also identified by non-reducing sodium dodecyl sulfate–polyacrylamide electrophoresis (SDS-PAGE) and NIRF imaging using the IVIS Spectrum CT imaging system (PerkinElmer). For VBPc loading determination, the micelles were lyophilized, weighted, and then destroyed in DMSO. The dissolved VBPc was measured by UV absorption at 800 nm for the estimation of drug loading.

### Photothermal Heating and Photoacoustic Imaging in Vitro

T-MP with different concentrations of VBPc (10, 20, 50, and 100 μg mL^−1^) in 96-well plates was irradiated by 808 nm laser (2 W cm^−2^) for 5 min. Temperature increases were monitored using the Pt100 temperature probe (Testo, Shanghai, China), and the photothermal images were recorded using thermal imaging camera (Testo 890). The laser power (1, 1.5, and 2.5 W cm^−2^)-dependent temperature increase was evaluated with 20 μg mL^−1^ VBPc-contained T-MP. The photothermal stability was also examined using 20 μg mL^−1^ of T-MP (200 μL) under 2 W cm^−2^ laser irradiation for three cycles. Photothermal conversion efficiency was calculated according to methods described in the literature [27–29].

For photoacoustic (PA) imaging evaluation, T-MP suspensions were immobilized in the polyurethane tubings (0.015 ID × 0.033 OD). A Vevo LAZR-X multimodal imaging system (Fujifilm VisualSonics) was used to acquire the PA images at an excitation of 770 nm. Different concentrations (2, 5, 20, 50, and 100 µg mL^−1^ VBPc) of T-MP were tested. The quantified PA signal intensity within region of interest (ROI) of each image was analyzed using the LAZR software (Fujifilm VisualSonics).

### Cellular Uptake and Photothermal-Induced Cytotoxicity

HT-29 cells were cultured in 96-well plate (1 × 10^4^ cells per well). When the cells reached about 80% confluence, the culture medium was replaced with 200 μL iFluor 594-labeled micelles (iFluor 594 0.1 μg mL^−1^) for 1 and 4 h, respectively. Then, the cells were fixed with a 4% paraformaldehyde solution for 15 min, and the cell nuclei were stained with 100 ng mL^−1^ Hoechst 33,342 for 8 min. The cellular uptake was assayed by quantifying the intracellular fluorescence intensity on a Thermo Scientific ArrayScan XTI High Content Analysis Reader (Thermo Fisher Scientific Cellomics, the USA). In another assay, the cells were seeded on the coverslip in 24-well plate (1 × 10^5^ per well). The images of cellular uptake were obtained using confocal laser scanning microscopy (CLSM) (Leica TCS SP8, Germany; iFluor 594: *E*_x_ 563 nm, *E*_m_ 604 nm).

For the evaluation of photothermal-induced cytotoxicity, HT-29 cells were seeded in 96-well plates at the density of 1 × 10^4^ cells per well. After 24 h culture, the cells were incubated with 20 μg mL^−1^ VBPc-contained T-MP for 4 h. Then, the cells were incubated in fresh medium and irradiated with 808 nm laser (2 W cm^−2^) for 20 min. After 24 h, the cell viability was assayed using Cell Counting Kit-8 according to the manufacturer’s instructions or calcein-AM/PI dual-staining test as we previously described [30]. Laser only, nonT-MP alone, or T-MP alone were included as controls.

### Orthotopic Colorectal Cancer Model and LN and Organ Metastasis Profile

The orthotopic colorectal cancer model was established as described in our previous report [24]. Briefly, female BALB/c nude mice were anesthetized with ketamine and xylazine. A 2–3 cm abdominal midline incision was made to expose the mouse cecum. Then, 2 × 10^6^ HT-29-luc cells suspended in 50 μL culture medium were orthotopically inoculated into the subserosa layer of the cecum wall using 30gauge needle (Hamilton, Reno, NV). The injection site was pressed with a cotton swab for 1–2 min to prevent cell effusion. The cecum was placed back into the peritoneal cavity, and the abdominal wall and skin were sutured with 5–0 suture, respectively.

The tumor formation and growth with the time were monitored using the IVIS Spectrum CT imaging system (PerkinElmer). Typically, the mice were intraperitoneally injected with d-luciferin (150 mg kg^−1^, J&K Chemical, Shanghai, China). After 8 min, the mice were anesthetized and imaged under the imaging system to examine the bioluminescence.

For LN and organ metastasis profile monitoring, mice at different time points (15 d, 22 d, 29 d, 36 d) after tumor cell inoculation were sacrificed. The LNs at three anatomical positions in the peritoneal cavity (pancreaticoduodenal, mesenteric, and lumbar LNs) and major organs (heart, liver, spleen, lung, and kidney) were excised for ex vivo bioluminescence imaging to examine the metastasis.

### Metastatic Mesenteric Sentinel LN Targeting and Biodistribution of T-MP

Cy5.5-labeled T-MP (Cy5.5 0.1 mg kg^−1^) was i.v. injected to HT-29-luc orthotopic tumor-bearing mice. After 2, 4, 8, and 24 h, respectively, the mice (*n* = 3 per group) were sacrificed, and the mesenteric sentinel LN was excised for fluorescence imaging assay using the IVIS Spectrum CT imaging system (*E*_x_ 685 nm, *E*_m_ 710 nm). The Cy5.5-labeled nonT-MP was included as control. The fluorescence intensities in the LNs were quantified and compared. LN targeting was also identified using PA imaging. The tumor-bearing mice (*n* = 3 per group) were i.v. injected with the micelles (VBPc 5 mg kg^−1^). After 8 h, the mesenteric sentinel LNs were excised for PA imaging, and the PA signal intensities of T-MP and nonT-MP in the LNs were recorded and compared. To examine the micro-distribution of the micelles, the LNs were processed for frozen section. The metastases (green; *E*_x_ 488 nm, *E*_m_ 520 nm) were stained using rabbit anti-HER2 antibody and FITC goat anti-rabbit secondary antibody. The image was obtained using Zeiss Axio Scan.Z1 slide scanner. The distribution of Cy5.5 labeled micelles (red; *E*_x_ 633 nm, *E*_x_ 710 nm) in the metastasis and non-metastasis region was observed and analyzed using ZEN 2.3 slidescan software.

For biodistribution study, mice at 8 h after injected with Cy5.5-labeled micelles were sacrificed. The tumor and major organs (heart, liver, spleen, lung, and kidney) were excised for fluorescence imaging assay in the IVIS Spectrum CT imaging system.

For VBPc assay, 50 mg of tissue or the weighted mesenteric sentinel LNs were homogenized with 3 mL 2% SDS. Then, 500 μL of the homogenate was added with 500 μL of methanol and chloroform (v/v 1:2) for 1 min vortex. The mixture was then centrifuged (5000*g*, 20 min), and the supernatant was taken to detect the absorption at 808 nm for VBPc determination.

### Photothermal Therapy of Metastatic Mesenteric Sentinel LNs and Antitumor Evaluation

Female BALB/c nude mice (*n* = 5) bearing orthotopic colorectal tumor were divided into six groups as following: (1) Untreated, (2) Tumor resection, (3) nonT-MP, (4) T-MP, (5) nonT-MP + Laser, and (6) T-MP + Laser. For group (3), (4), (5), and (6), tumor resection was also performed. The micelles with VBPc 5 mg kg^−1^ for each mouse were i.v. injected to the mice when involved. 8 h after injection, the orthotopic tumor was resected, and the mesenteric sentinel LN was irradiated using 808 nm laser (2 W cm^−2^, 10 min). An infrared thermal camera (Testo 890) was used to monitor the temperature change of the LN during the laser irradiation. Then, the abdominal wall and skin were sutured. To examine the effect on the LN microstructure, the LNs were excised for ultrathin section making and observed under TEM [31].

The tumor growth and recurrence were monitored by bioluminescence imaging every 7–8 days. The mouse survival time and body weight were monitored every other day post various treatments. Increase in life span (*ILS*) was calculated using the formula: %ILS = (*T*/*C* − 1) × 100% [32]. *T* and *C* were the median survival of the tumor-bearing mice in the treated and untreated groups, respectively.

### Histopathological and Immunohistochemical Assay

To identify the LN metastases, the mesenteric sentinel LNs of the mice were excised and processed for hematoxylin and eosin (H&E) staining and pathological examination. The HER2-positive metastases in LNs and the specimens of human colorectal cancer were also demonstrated through rabbit anti-HER2 antibody immunohistochemical staining.

To investigate the toxicity of micelles on the organs, 24 h after i.v. injection of micelles (VBPc 5 mg kg^−1^, *n* = 3), major organs (heart, liver, spleen, lung, and kidney) of the tumor-bearing mice were excised and processed for H&E staining and pathological examination under photomicroscope (Nikon Eclipse E200).

### Blood Analysis

Healthy female BALB/c nude mice were i.v. injected with micelles (VBPc 5 mg kg^−1^). After 24, 96, and 169 h, respectively, retro-orbital blood was collected for serum biochemistry and complete blood panel analysis, which were performed in Drug Safety Evaluation Research Center (Shanghai Institute of Materia Medica, Shanghai, China). Three mice were included for each group.

### Statistical Analysis

Statistical analysis was performed using GraphPad Prism 8.0 software (La Jolla, CA). Differences between groups were assayed using Student’s *t*test or ANOVA with Tukey’s multiple comparison tests. The p value below 0.05 was considered significant.

## Results and Discussion

### Preparation and Characterization of Targeted Micellar Phthalocyanine (T-MP)

Pluronic F127, an FDA-approved non-ionic copolymer widely used for drug delivery [33], was adopted for the preparation of micellar phthalocyanine as previously described [19]. For trastuzumab modification, the terminal hydroxyl group of F127 was carboxylated via reaction with succinic anhydride [25]. A C=O stretching signal at 1737.3 cm^−1^ in the FTIR spectrum indicated successful carboxylation of Pluronic F127 (Fig. S1).

Empty F127 micelles were formed through dispersing F127 (15% carboxylation) in water (10% w/v). Then, 2 mg VBPc (structure shown in Fig. S2) in 1 mL dichloromethane was added. The mixture was stirred until the dichloromethane was evaporated to generate VBPc-loaded F127 micelles. The micelles were then purified via CMC switching and centrifugal filtration at 4 °C to remove the empty micelles and loosely bound F127 to provide the resulting concentrated micellar phthalocyanine (MP) [19]. Trastuzumab was then linked to MP surface via EDC/NHS chemistry to obtain targeted micellar phthalocyanine (T-MP). Trastuzumab modification conferred two amide peaks at 1644 cm^−1^ and 1563 cm^−1^ in the FTIR spectra (Fig. S1) and also increased surface nitrogen (17.9%) compared to that (3.7%) of the unmodified MP identified by X-ray photoelectron spectroscopy (XPS) (Fig. S3).

**Fig. 1:**
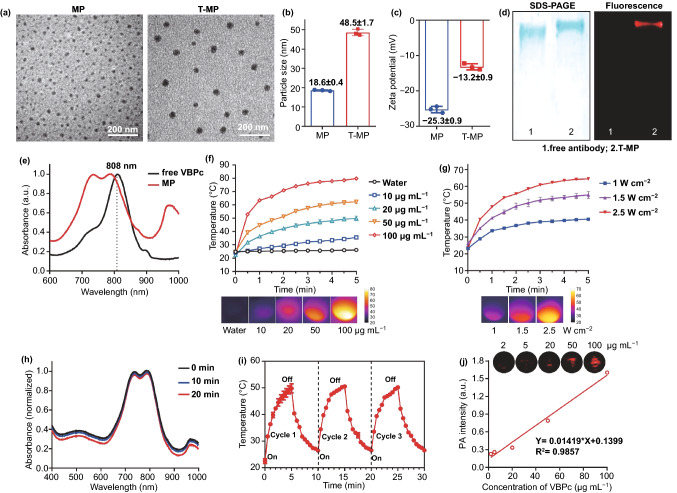
Characterization of micellular VBPc. **a** TEM images of MP (unmodified) and T-MP. Particle size (**b**) and zeta potential (**c**) determined by dynamic light scattering (DLS). **d** SDS-PAGE with coomassie blue staining (left) and near-infrared fluorescence imaging (right) of free antibody and T-MP. **e** Vis–NIR absorbance of free VBPc in dichloromethane and unmodified MP in water. **f** VBPc concentration-dependent temperature increases of T-MP at power density of 2 W cm^−2^. Representative photothermal images at 5 min were captured by thermal imaging camera (Testo 890) **g** Light power-dependent temperature increase curves of T-MP (20 μg mL^−1^ VBPc). Representative photothermal images at 5 min were shown. **h** Consecutive cycles of laser-induced heating of T-MP (VBPc 20 μg mL^−1^) under 808 nm irradiation at 2 W cm^−2^. **i** Influence of irradiation time on the Vis–NIR absorption curve of T-MP. **j** PA imaging property of T-MP. Data are presented as mean ± s.d. (*n* = 3)

Unmodified MP was 20 nm spheres, and antibody modification (T-MP) increased the size to 40 nm as observed in transmission electron microscopy (TEM) (Figs. 1a and S4). This size change was also confirmed in the dynamic light scattering (DLS) (Fig. 1b), showing a hydrodynamic size of 48 nm for T-MP versus 18.6 nm for unmodified MP. Trastuzumab modification decreased the nanoparticle surface charge from − 25.3 to − 13.2 mV (Fig. 1c), which may be ascribed to the depletion of the carboxyl groups of the F127 micelles.

Non-reducing sodium dodecyl sulfate–polyacrylamide gel electrophoresis (SDS-PAGE) with coomassie blue staining assay was also performed to examine the antibody modification. Compared to the free antibody, T-MP moved slightly more slowly due to the increased size, although the molecular weight of F127 (12.7 kDa) is significantly less than the antibody (~ 150 kDa) (Fig. 1D). VBPc fluorescence in the micelles was largely self-quenched due to their aggregation in micelle core (Fig. S5). For micelle fluorescent labeling, Cy5.5-NHS ester that could be reacted with the antibody amine groups was incubated with T-MP to generate Cy5.5-labeled T-MP. The corresponding single Cy5.5 signal band indicated the successful fluorescent labeling, high T-MP purity, and high conjugation efficiency (~ 100%) of antibodies (Fig. 1D). We further determined the number of antibodies on each nanoparticle. Through BCA assay, the antibody loading in T-MP was estimated to be 1.5%. The weight of each T-MP particle (m) was calculated using the equation: *m* = ρ*(πD^3^/6) [34]. In this equation, *ρ* was the nanoparticle weight per volume unit (density), estimated to be 1 g cm^−3^. D was the number-based mean nanoparticle diameter (48 nm) determined by DLS. Thus, the number of antibodies on each nanoparticle was estimated to be 28.

Free VBPc had a maximum absorption peak at 808 nm, while the micellar VBPc had absorption peaks of comparable intensity at 736 and 790 nm (Fig. 1e), consistent with previous reports [19]. After disrupting the lyophilized micelles with DMSO, VBPc loading in T-MP was determined to be 69.1% using the optical absorbance at 800 nm. This significantly high drug loading, compared to that (typically below 5%) [35] produced using the conventional method without stripping process, reflected the advantage of micelle production using the surfactant-stripping strategy [36, 37]. High drug loading would generally decrease the excipient-associated side effect and may also enable higher dose of drug to be used, which can be useful when drugs need to be administered in a smaller volume [36]. Upon 808 nm light irradiation (2.0 W cm^−2^, 5 min), micellar VBPc in water exhibited concentration-dependent (10–100 μg mL^−1^) temperature increase to 79.9 °C (Figs. 1f and S6). Laser power (1–2.5 W) -dependent temperature elevation of T-MP (20 μg mL^−1^ VBPc) was also characterized (Figs. 1g and S7). T-MP demonstrated good photothermal stability; after 20 min laser irradiation, the optical absorption spectrum and intensity remained nearly unchanged (Fig. 1h). Also, three consecutive cycles of NIR-induced heating achieved the similar and comparable temperature increase patterns (Fig. 1i). The photothermal conversion efficiency of T-MP was calculated to be 16.8%, which was comparable to that of Au nanorod (17%) [17], but was lower than the emerging semiconducting polymer nanoparticles (27.5 ~ 71%) [17, 27, 38, 39]. Micellar VBPc also possessed strong photoacoustic (PA) imaging properties (Fig. 1j), which can be used to identify the distribution in the metastatic LN, as well as for photothermal contrast. No obvious differences were observed with respect to the optical properties of nanoparticles before and after antibody conjugation (Fig. S8), indicating the minimal influence of antibody conjugation on the micellular core where VBPc located. T-MP was colloidally stable in PBS at 4 ℃ for 7 days, PBS with 10% FBS at 37 ℃ for 48 h, and McCoy's 5A medium at 37 ℃ for 48 h with the size remained almost unchanged (Fig. S9).

### Enhanced Uptake and Photothermal Cytotoxicity of T-MP in HT-29 Cells

**Fig. 2:**
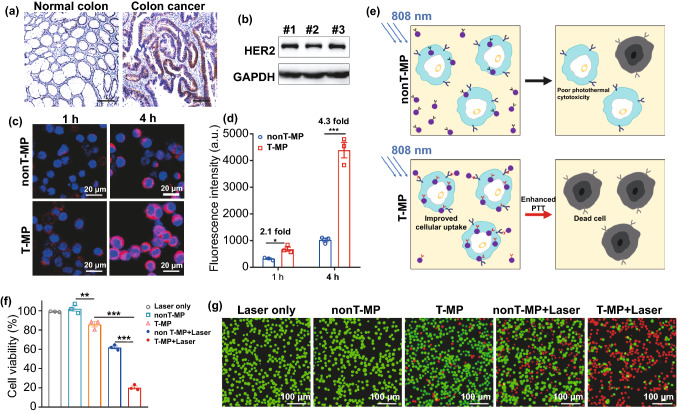
Targeted cellular uptake and improved photothermal-induced cytotoxicity. **a** Representative immunohistochemical staining showing high HER2 expression in colon cancer from human patients, compared to human normal colon tissues. **b** Western blot analysis for HER2 expression in HT-29 cells. #1, #2, and #3 are three replicated samples. GAPDH (glyceraldehyde-3-phosphate dehydrogenase) was used as control. **c** Confocal fluorescence images of cellular uptake in HT-29-luc cells after 1 and 4 h-incubation with iFluor 594-labeled micelles (*E*_x_ 563 nm, *E*_m_ 604 nm). **d** iFluor 594 fluorescence intensity in HT-29 cells analyzed by ArrayScan XTI High Content Analysis Reader. **e** Illustration showing how improved cellular uptake conferred by targeted antibody modification enhances photothermal-induced cytotoxicity. **f** Cell viability after treatment with micelles and laser irradiation. Micelles or laser alone were included as control. **g** Fluorescence microscope images of calcein-AM/PI co-stained HT-29-luc cells after various treatments. Live and dead cells were shown in green (Calcein-AM; *E*_x_ 490 nm, *E*_m_ 515 nm) and red (PI; *E*_x_ 530 nm, *E*_m_ 580 nm), respectively. Data are presented as mean ± s.d. (*n* = 3). *p < 0.05, **p < 0.01, ***p < 0.001

HER2 is a clinically relevant therapeutic target for multiple cancers, including breast, gastric, and lung cancer [40]. HER2 is also found to be involved in the progression of some colorectal cancers, and anti-HER2 therapy against colorectal cancer is undergoing in clinical trials [41, 42]. Indeed through immunohistochemical assay of the tumor tissues from 13 colorectal cancer patients, we also identified that HER2 expression in tumor cells was higher than that in the tumor stroma and adjacent normal tissues (Figs. 2a and S10). These observations suggest that the HER2 receptor may be a clinically relevant target, at least for a portion of patients, for colorectal cancer-targeted drug delivery. HT-29, a human colorectal cancer cell line with strong HER2 expression (Fig. 2b) [22, 23], was used in this study. Micelles labeled with iFluor 594 were used for the cell uptake assay. Compared with non-targeted isotype lgG-conjugated micelles (nonT-MP), trastuzumab modification (T-MP) enhanced cellular uptake by 2 ~ 4 times in a time-dependent manner (Fig. 2c, d). This effect would lead to enhanced photothermal cytotoxicity to HT-29-luc cells as illustrated in Fig. 2e. In another test, HER2-negative 4T1 cells were also used for cellular uptake assay (Fig. S11). No differences were observed between the uptake of T-MP and nonT-MP in 4T1 cells. This evidence together with the observation in HT-29 cells demonstrated the specific targeting ability of T-MP in HER2-positive cells.

For photothermal therapy in vitro, HT-29-luc cells were treated with micellular VBPc then subjected to 808 nm laser irradiation. As expected, the photothermal killing effect caused by T-MP (only 20.3% viable cells left) was stronger than that of nonT-MP (61.9% cell viability) (Fig. 2f). No cytotoxicity occurred to the cells treated with nonT-MP alone or laser alone. However, trastuzumab attached to the micelle surface exhibited moderate killing effect on HT-29 cells; this can be ascribed for the blockage of the HER2 signaling pathway [40].

The improved photothermal therapy conferred by T-MP was further confirmed by calcein-AM and propidium iodide (PI) dual-staining tests (Fig. 2g). The least live (green) and most dead (red) cells after T-MP targeted photothermal treatment indicated the strongest cytotoxic killing.

### Orthotopic Colorectal Cancer Model and Targeted Accumulation of T-MP in Metastatic Sentinel LNs

**Fig. 3:**
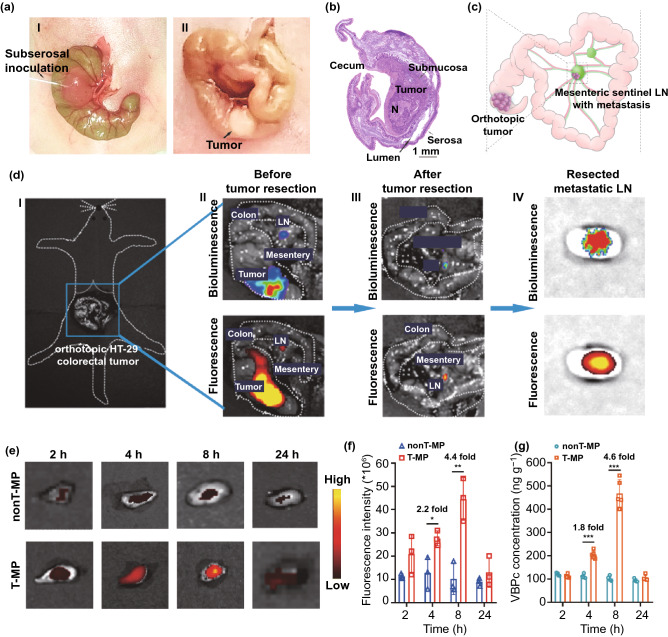
Orthotopic HT-29-luc colorectal cancer model and metastatic LN targeting of T-MP. **a** Establishment of orthotopic HT-29-luc tumor by sub-serosal injection of tumor cells in the cecum (I). The solid tumor was visible to the naked eye after 2 weeks (II). **b** Hematoxylin and eosin (H&E) staining showed the right anatomical location of the orthotopic tumor. **c** Illustration of the metastatic sentinel LN in the mesentery. **d** The metastatic mesenteric sentinel LN was targeted and imaged using Cy5.5-labeled T-MP after intravenous administration. (I), Illustration of the orthotopic HT-29-luc tumor in mouse abdominal cavity. (II), Bioluminescent and fluorescent imaging of the orthotopic tumor and metastatic sentinel LN. (III), Bioluminescent and fluorescent imaging of the metastatic sentinel LN after tumor resection. (IV), Ex vivo imaging of the excised metastatic sentinel LN. **e** Time-dependent, ex vivo imaging of the Cy5.5-labeled micelles in the metastatic sentinel LNs. **f** Quantified fluorescence intensity in panel E. **g** VBPc contents in metastatic sentinel LN. Data are presented as mean ± s.d. *n* = 3 in panel F and 5 in panel G. *p < 0.05, **p < 0.01, ***p < 0.01

With injection of tumor cells into submucosa of cecum, the orthotopic HT-29-luc colorectal cancer model was established (Fig. 3a, b) [24]. HT-29-luc tumor growth was accompanied by LN metastasis that mostly occurred in the mesentery as illustrated in Fig. 3c. Pathological and immunohistochemical analysis showed that the detectable metastases were usually solitary and located in the LN cortex region (Fig. S12).

Through sensitive bioluminescence imaging showing that as least as 100 tumor cells can also be detected (Fig. S13), we identified the LN metastasis pattern with time. Within 15–36 days after tumor cell inoculation, LN metastasis only occurred to the mesenteric LN, but not the pancreaticoduodenal LNs, and the lumbar LNs (Fig. S14). It is noted that during day 15 to 22, metastasis only happened to the sentinel LNs, but not the next ones following the flow of LN direction. Meanwhile, no organ metastasis appeared within 22 days after tumor cell inoculation (Fig. S15). Therefore, mice with tumor cell inoculation for 15–22 days were used in this study.

It has been well demonstrated that small-sized nanoparticles (< 50 nm), but not large ones, can effectively enter into the LN metastases to enable therapy [7]. Based on this finding, we here explored whether such nanoparticle accumulation in LN metastasis could be further improved by endowing the nanocarriers with the targeting property for tumor cells. Mice bearing orthotopic tumors and LN metastases were then intravenously administered with Cy5.5-labeled T-MP. As demonstrated in Fig. 3d, T-MP successfully targeted to the metastatic mesenteric sentinel LN as demonstrated by sequential bioluminescence and fluorescence imaging in situ or ex vivo. We further examined the time-dependent distribution of T-MP in the metastatic LNs (Fig. 3e, f). The accumulation of T-MP increased with the time and reached its peak at 8 h after injection. Importantly, the targeting efficacy of T-MP in the metastatic sentinel LNs was markedly higher than that of nonT-MP, exhibiting 2.2- and 4.4-folds increased distribution at 4 and 8 h, respectively. This advantage may be ascribed to the small micelle size and the improved tumor cell targeting and uptake conferred by the conjugated anti-HER2 antibody (Fig. 2c, d).

The biodistribution of the micelles and the cargo (VBPc) at 8 h after injection was examined. The accumulation of T-MP in tumor tissue was higher than that of nonT-MP (Fig. S16), and same observation was obtained for the VBPc contents in the tumors (Fig. S17). There was no significant difference in their distribution in other organs. To examine whether the enhanced distribution in the primary tumor contributed to the improved accumulations in metastatic sentinel LN, in another test, the orthotopic tumor was in advance excised before the micelle injection. It showed that the distribution of T-MP in metastatic sentinel LNs was comparable to that without primary tumor resection, and higher than that of nonT-MP (Figs. S18 and 3e, f). Moreover, the micelle distribution in healthy LNs was obviously much lower. These observations were consistent with the literature, demonstrating that metastatic LN targeting by small-sized nanoparticles was less relevant to primary tumor [7]. This improved micelle distribution led to elevated VBPc contents in metastatic sentinel LNs (Fig. 3g), which would confer stronger photothermal effects when light irradiation was performed.

**Fig. 4:**
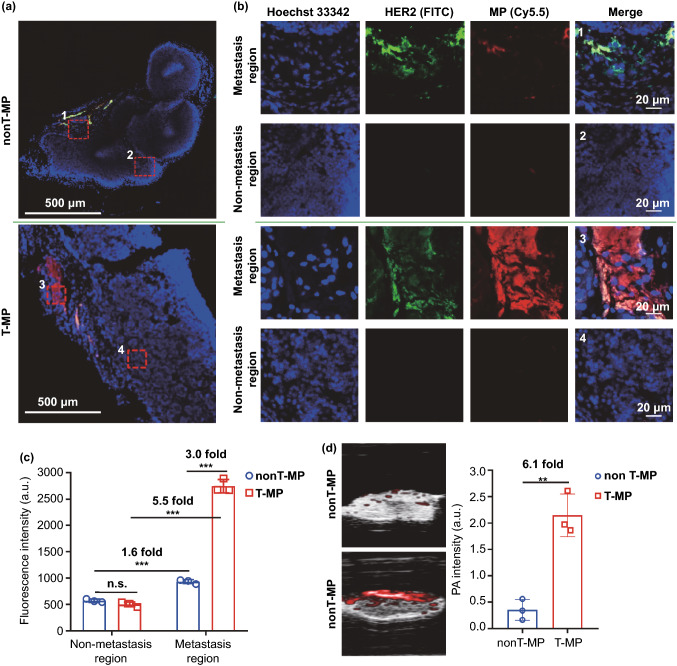
Micro-distribution of the micelles in the metastatic sentinel LNs and LN PA imaging. **a** Immunofluorescent staining of the metastatic sentinel LNs 8 h after i.v. injection of the T-MP or nonT-MP. The micelles were labeled with Cy5.5 (red) and the metastatic tumor cells were stained with rabbit anti-HER2 antibody and FITC goat anti-rabbit antibody (green). The region with metastasis (1 and 3) and metastasis-free region (2 and 4) was enlarged in panel (**b**). **c** Quantified fluorescence intensity in panel B. n.s., not significant. **d** Ex vivo PA imaging of the excised metastatic sentinel LNs. Quantified PA signals were compared. Data are presented as mean ± s.d. (*n* = 3). **p < 0.01, ***p < 0.001

We further performed immunofluorescence staining of the metastatic LNs to investigate the micro-distribution of micelles in the LNs and the targeting of Cy5.5-labeled micelles (red) to the metastatic foci (anti-HER2 staining, green) (Fig. 4a, b). The micelles were found to be mainly distributed in the metastasis region, and the accumulation of T-MP in metastasis region was 3.0-folds higher than that of nonT-MP micelles (Fig. 4c). There was no significant difference between the low-distribution of the two micelles in the non-metastasis region of the LNs. Noted that the accumulation of nonT-MP in metastasis region was 1.6-fold higher than that in the non-metastasis region, demonstrating the LN metastasis targeting capacity of small-sized particles [7].

PA imaging also showed that compared with nonT-MP, the PA signal of T-MP in metastatic LNs was much higher with around 6 times enhancement (Fig. 4d). Noted that the PA signal was unevenly distributed in the LNs, but specifically distributed more on one side of the LNs. This phenomenon was consistent with the observation that the metastatic foci were located at peripheral cortex region of the LNs (Fig. S12).

### Photothermal Therapy of Metastatic LNs and Antitumor Efficacy

**Fig. 5:**
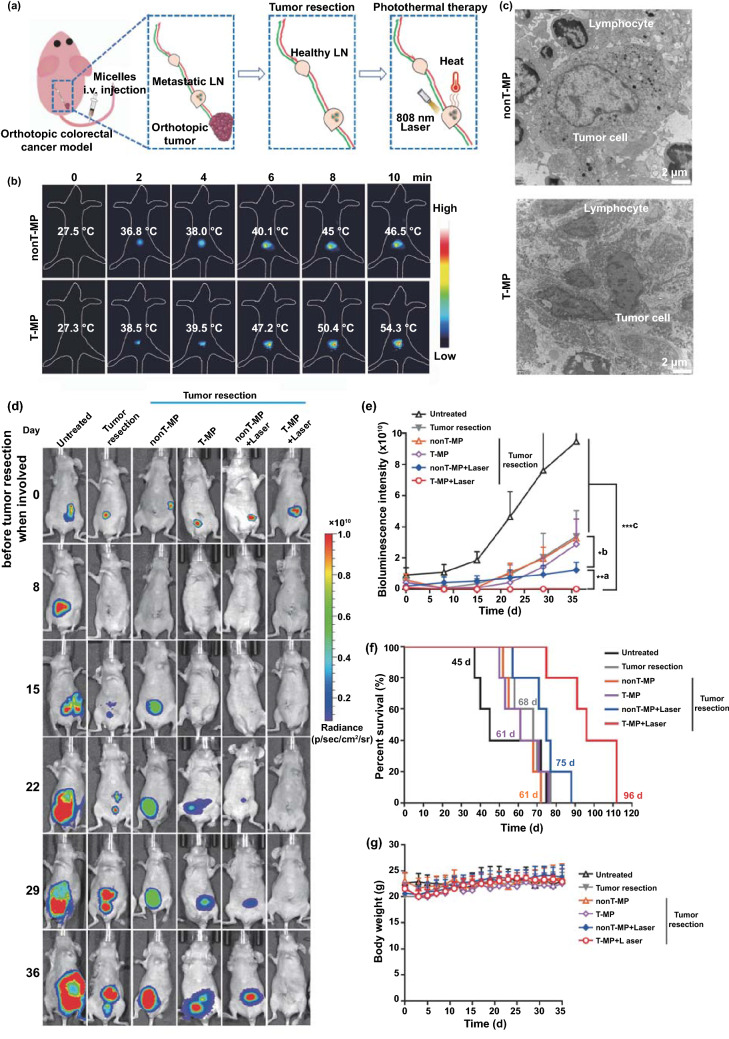
Photothermal therapy of metastatic sentinel LNs and antitumor evaluation. **a** Illustration of the treatment of the surgical resection of orthotopic tumor and follow-up laser irradiation of the metastatic LNs on day 0. **b** Temperature increase of the metastatic LNs was recorded using Testo 890 thermal imager. **c** Representative TEM images of LN sections after photothermal therapy. **d** Representative bioluminescence imaging of the mice before tumor resection (day 0) and after tumor resection and photothermal therapy over the time. **e** Quantified bioluminescence intensity in panel D. (1) T-MP + Laser versus nonT-MP + Laser; (2) nonT-MP + Laser versus T-MP, nonT-MP, and Tumor resection. (3) T-MP + Laser versus all other controls except nonT-MP + Laser. **f** Survival of the mice with indicated treatments. The median survivals of the mice were noted. **g** Mice body weight. Data are presented as mean ± s.d. (*n* = 5). *p < 0.05, **p < 0.01, and ***p < 0.001

We next investigated the benefit of photothermal therapy of metastatic LNs conferred by T-MP over nonT-MP. The HT-29-luc tumor-bearing mice were i.v. injected with T-MP or nonT-MP (5 mg kg^−1^ VBPc). After 8 h, the orthotopic tumor was surgically resected, and the sentinel LN was irradiated with 808 nm laser (2 W cm^−2^, 10 min) for thermal ablation of the metastatic tumor cells. The experimental procedure is illustrated in Fig. 5a. The temperature of the sentinel LN treated with T-MP increased from 27.3 to 54.3 °C, pronounceably higher than that (46.5 °C) treated with nonT-MP (Fig. 5b). This observation could be ascribed to the improved distribution of T-MP compared to nonT-MP in the metastatic LN (Fig. 3e–g). Moreover, this higher temperature might cause enhanced killing to the tumor cells in the LN. After 48 h, the irradiated sentinel LNs were excised and processed for electron microscopic observation. More dead tumor cells characterized by cell shrinkage, nuclear cracking and pyknosis were observed in the metastatic LNs treated with T-MP, but not nonT-MP, assisted photothermal therapy (Fig. 5c).

The untreated mice or the mice treated with tumor resection only or together with the micelles but no laser irradiation were included as controls. The growth or recurrence of the tumors and the mice survival as well as body weight were monitored and recorded. It showed tumor recurrence was dramatically delayed and suppressed when the metastatic LNs were treated with T-MP-assisted laser irradiation (Fig. 5d, e). Accordingly, mice life span was dramatically extended with median survival of 96 d, earning a biggest increase in life span (*ILS*) of 113.3%, compared with other controls (Fig. 5f, Table S1). It is noted that this therapeutic outcome was comparable to or even better than the traditional treatment of tumor resection plus lymphadenectomy, which earned a 91-day median survival (Fig. S19), demonstrating the advantage of this local and minimally invasive treatment. In contrast, lymphadenectomy usually causes several risks and complications such as bleeding, infection, pain, phlebitis, nerve injury, and lymphedema, which compromise the quality of life [43].

To determine the cause of mouse death, we performed autopsy of the mice from T-MP + Laser group through bioluminescence imaging. The luciferase in the tumor cells was still active shortly after the mouse death. For bioluminescence imaging, the resected organs were previously soaked with the PBS containing luciferin, Mg^2+^, and ATP for 5 min as we described [44]. Tumor recurrence in situ (intestine) or the metastases in liver and lung were observed in the mice of both T-MP + Laser group (Fig. S20) and nonT-MP + Laser group (Fig. S21), which would be responsible for the animal death. The metastases in the organs were also confirmed using hematoxylin and eosin (H&E) staining (Figs. S22 and S23).

Following treatment, the mouse body weights were well maintained (Fig. 5g), indicating the tolerance of the treatment. Pathological examination of the organs, including heart, liver, spleen, lung, and kidney in all groups showed no significant histological toxicity (Fig. S24). Blood analysis was also performed for the treatment-associated toxicity evaluation. Key parameters reflecting liver and kidney function and hematological toxicity were examined after 24, 96, and 168 h (Figs. S25–S27). It showed no obvious difference in the parameters of T-MP plus laser group, when compared to other controls, indicating the low toxicity of the therapy.

## Conclusions

In summary, this study proposed a new method to improve the targeting and treatment of LN metastases. Through introducing targeting capacity, small-sized nanoparticles (< 50 nm) accumulated more in LN metastasis than the non-targeted counterparts, as proved using a metastatic orthotopic colorectal tumor model. This improved targeting could be used for enhanced therapy such as local and minimally invasive photothermal therapy to overcome LN metastasis and suppress disease recurrence, eventually leading to a prolonged animal survival. Moreover, the improved therapeutic outcome of the metastatic LNs was comparable or even better than the traditional lymphadenectomy that usually incurred surgical trauma and postoperative complications. Our findings exhibited the potential of targeted small-sized nanoparticles to enable superior paradigms for dealing with LN metastases.

## Supplementary Information

Below is the link to the electronic supplementary material.

Supplementary file1 (PDF 3751 KB)
