# Supplementary material for: Targeted Micellar Phthalocyanine for Lymph Node Metastasis Homing and Photothermal Therapy in an Orthotopic Colorectal Tumor Model
Source: Nanomicro Lett. 2021 Jun 19;13:145. doi: 10.1007/s40820-021-00666-8 (PMC8214644; doi:10.1007/s40820-021-00666-8)
Supplement: Supplementary file 1 — Supplementary file1 (PDF 3751 KB) [file 40820_2021_666_MOESM1_ESM.pdf]

Nano-Micro Letters

Supporting Information for

Targeted Micellar Phthalocyanine for Lymph Node Metastasis Homing and Photothermal Therapy in an Orthotopic Colorectal Tumor Model

Hai-Yi Feng[1, 2, #], Yihang Yuan[1, #], Yunpeng Zhang[2], Hai-Jun Liu[1], Xiao Dong[1], Si-Cong Yang[1], Xue-Liang Liu[1], Xing Lai[1], Mao-Hua Zhu[1], Jue Wang[2], Qin Lu[1], Quanjun Lin[2], Hong-Zhuan Chen[3], Jonathan F. Lovell[4], Peng Sun[2, *], Chao Fang[1, *]

[1]Hongqiao International Institute of Medicine, Tongren Hospital and State Key Laboratory of Oncogenes and Related Genes, Department of Pharmacology and Chemical Biology, Shanghai Jiao Tong University School of Medicine (SJTU-SM), Shanghai 200025, P. R. China

[2]Department of General Surgery, Tongren Hospital, SJTU-SM, Shanghai 200336, P. R. China

[3]Institute of Interdisciplinary Integrative Biomedical Research, Shuguang Hospital, Shanghai University of Traditional Chinese Medicine, Shanghai 201203, P. R. China

[4]Department of Biomedical Engineering, University at Buffalo, State University of New York, Buffalo, NY 14260, USA

[#]Hai-Yi Feng and Yihang Yuan contributed equally to this work.

*Corresponding authors. E-mail: fangchao32@sjtu.edu.cn (C. Fang), sp2082@shtrhospital.com (P. Sun)

Supplementary Figures and Table

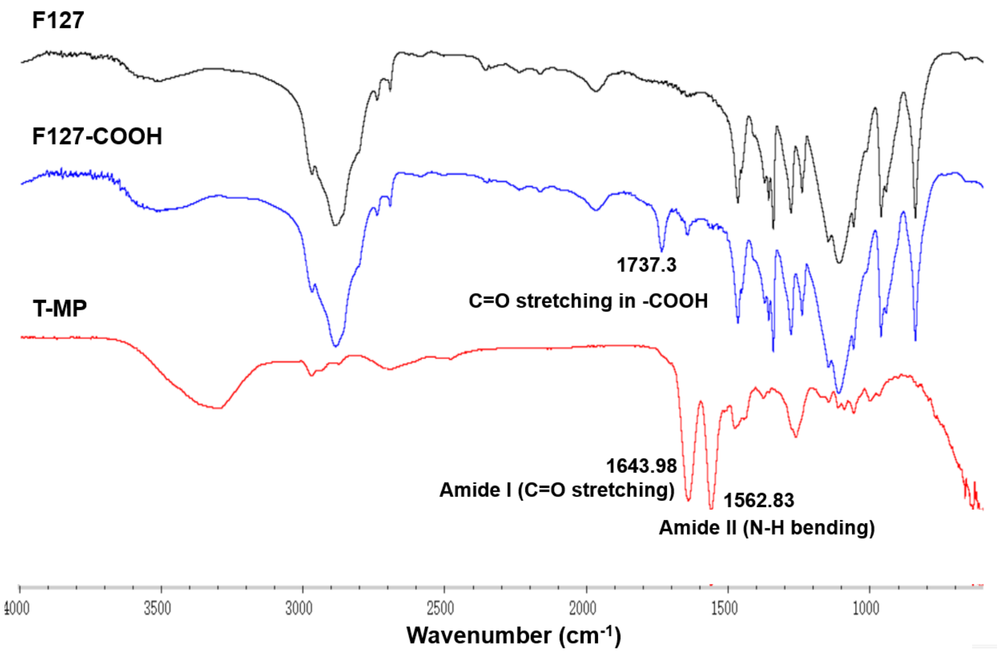

Fig. S1 FTIR spectrum of F127, carboxylated F127 (F127-COOH), and T-MP

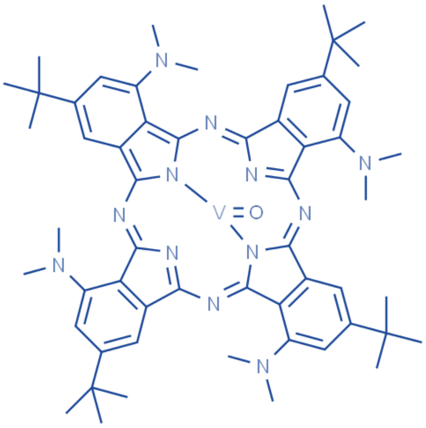

Fig. S2 Structure of Vanadyl 3,10,17,24-tetra-tert-butyl-1,8,15,22-tetrakis(dimethylamino)-29H,31H-phthalocyanine (VBPc) ($C_{56}H_{68}N_{12}OV$, MW 976.160)

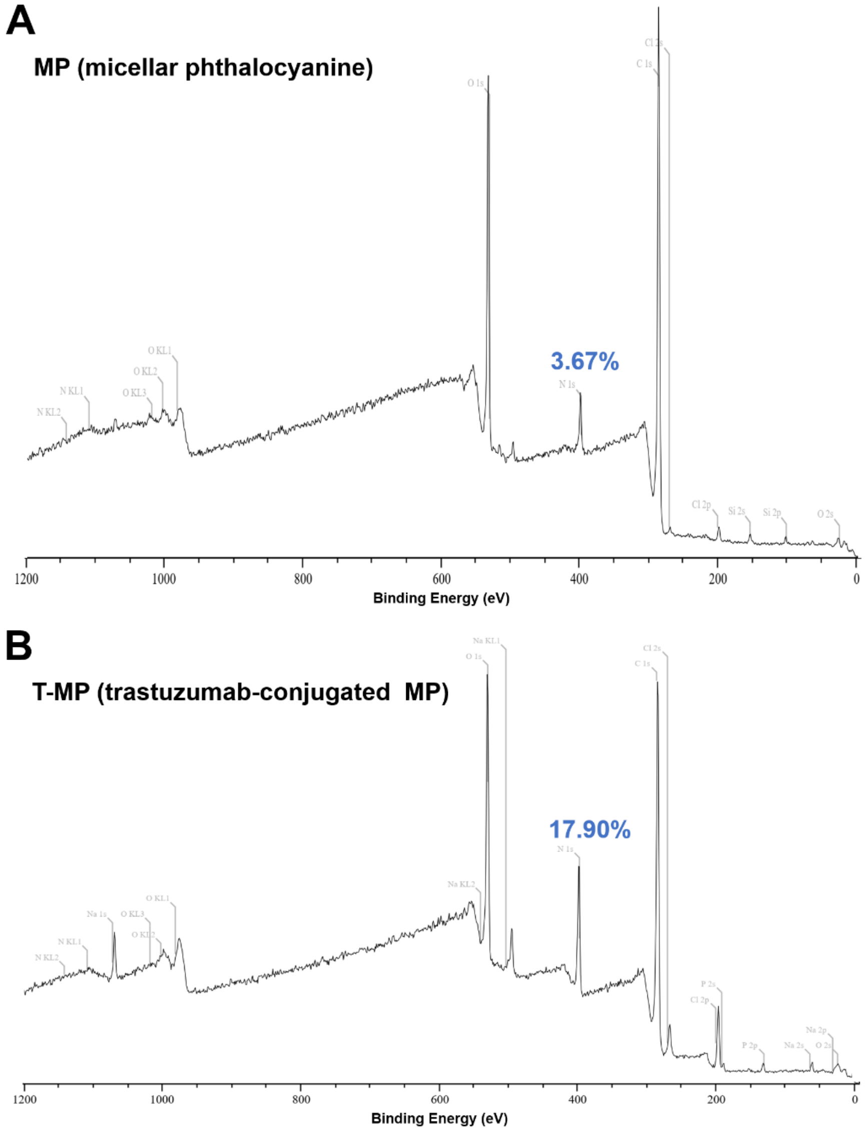

Fig. S3 X-ray photoelectron spectroscopy (XPS) assay of MP (micellar phthalocyanine) (**A**) and T-MP (trastuzumab-conjugated MP) (**B**). The surface nitrogen contents were noted

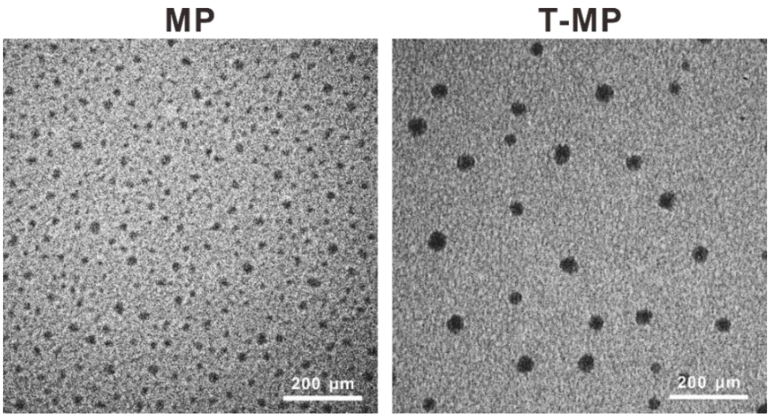

Fig. S4 Due the layout restriction in Fig. 1, amplified TEM images or fields of view of MP (unmodified) and T-MP are provided here

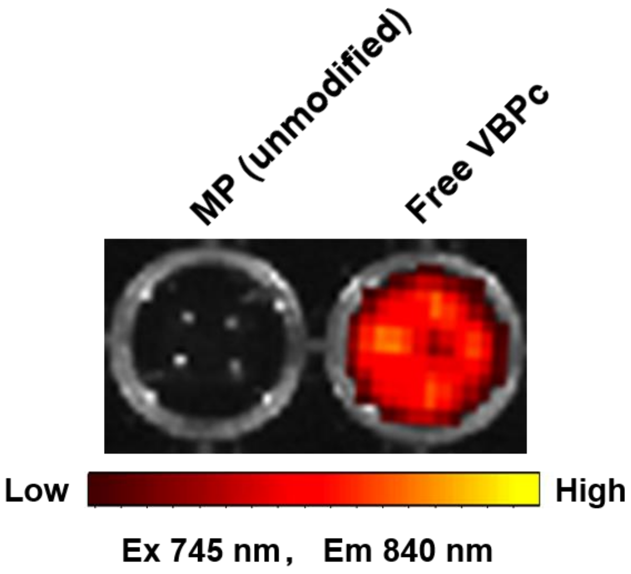

Fig. S5 Fluorescence of VBPc quenched when loaded in F127 micelles. Images were obtained using IVIS spectrum CT imaging system (E_x 745 nm, E_m 840 nm)

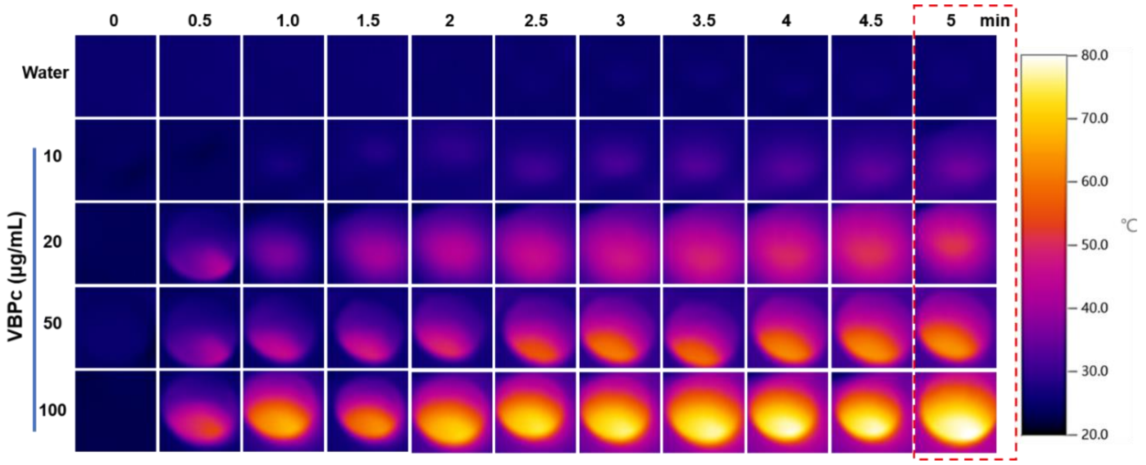

Fig. S6 Concentration-dependent temperature increase. VBPc in T-MP was 10, 20, 50, and 100 μg/mL in the 96 well plate. The power of 808 nm laser was 2 W/cm^2. Representative photothermal photos were shown. The contents in the red dotted line are also displayed in Figure 1F in the main text

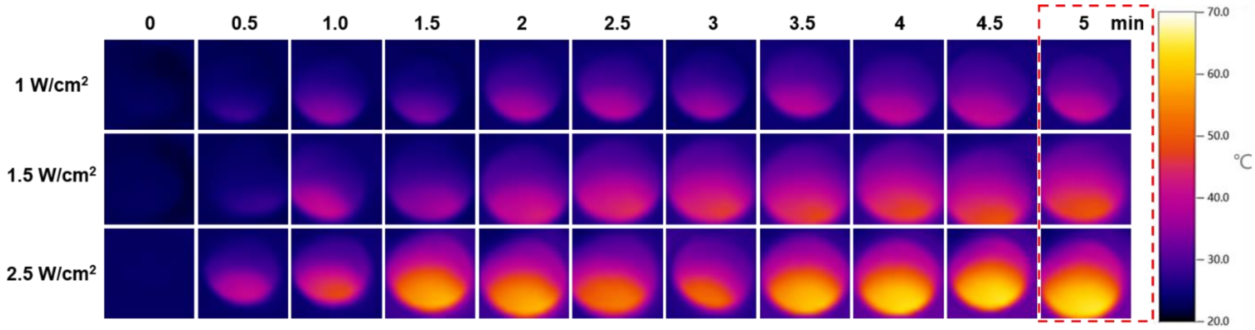

Fig. S7 Laser power-dependent temperature increase. VBPc in T-MP was 20 μg/mL in the 96 well plate. The power of 808 nm laser was 1, 1.5, and 2.5 W/cm^2. The contents in the red dotted line are also displayed in Figure 1G in the main text

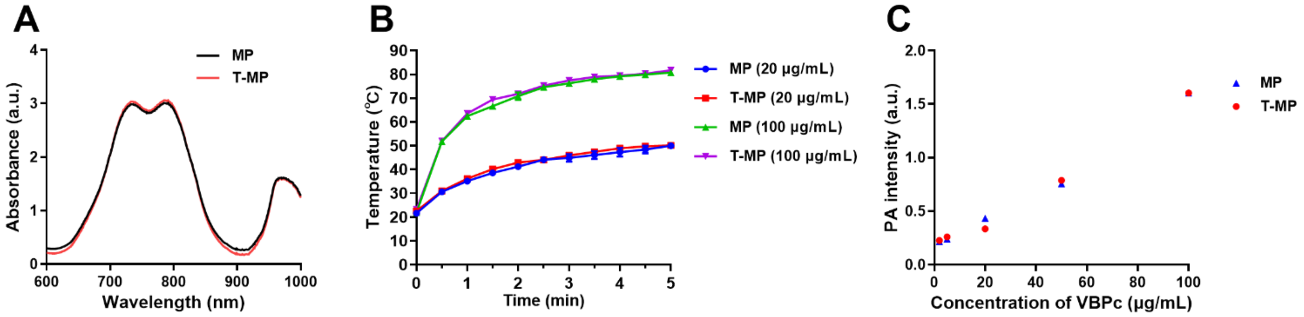

Fig. S8 Influence of antibody conjugation on the optical properties of nanoparticles. (**A**) Vis-NIR absorbance of MP and T-MP in water. VBPc concentration was 20 μg/mL. (**B**) Temperature increases induced by nanoparticles with laser power density of 2 W/cm^2. (**C**) PA intensity comparison of MP and T-MP. Data are presented as mean ± s.d. (n = 3)

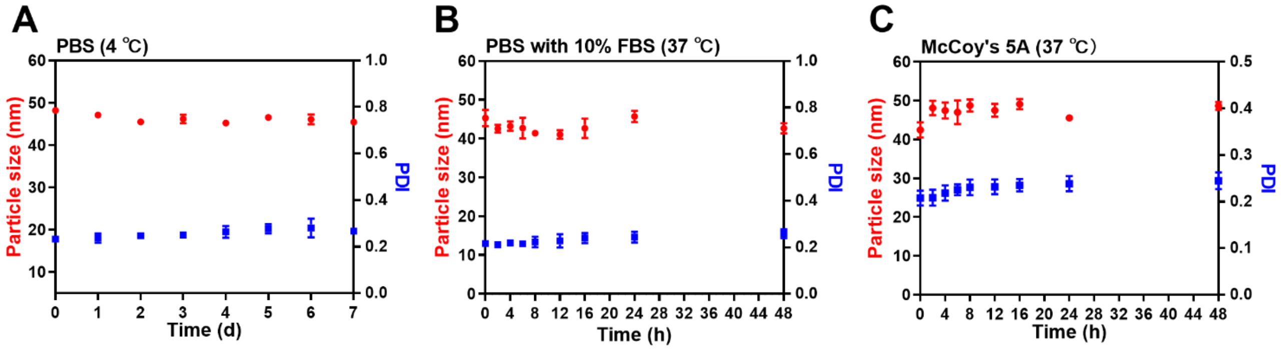

Fig. S9 Colloidal stability of T-MP in PBS at 4 °C (**A**), PBS with 10% FBS at 37 °C (**B**), and McCoy's 5A medium at 37 °C (**C**). Data are presented as mean ± s.d. (n = 3)

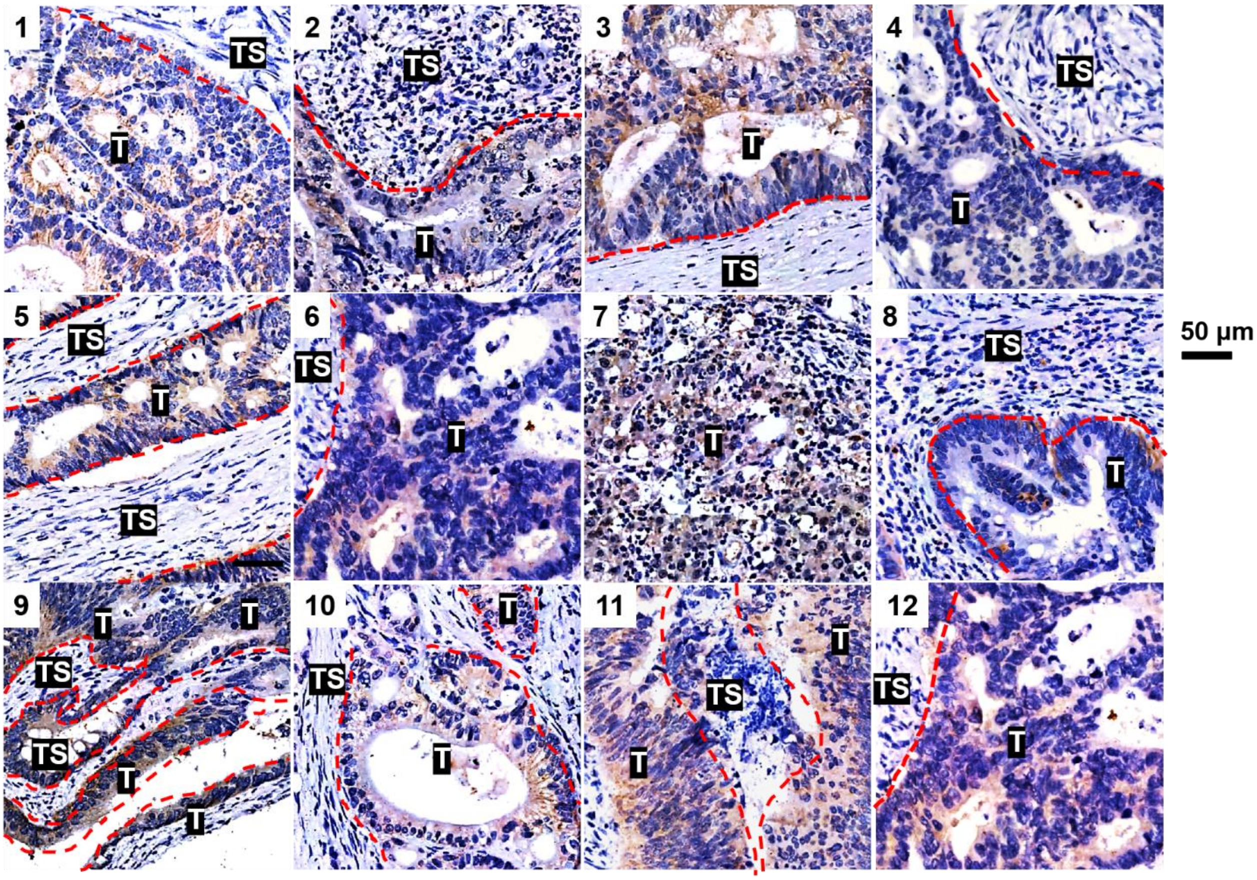

Fig. S10 Immunohistochemical staining of HER2 expression in the specimens of human colorectal cancer from 12 patients. T: tumor nest; TS, tumor stroma

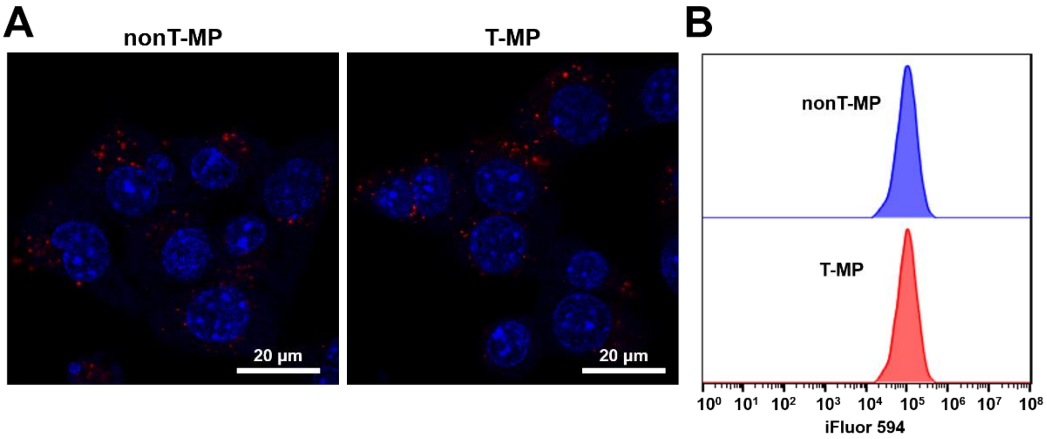

Fig. S11 Cellular uptake of micelles in 4T1 cells. (**A**) Confocal fluorescence images of cellular uptake in 4T1 cells after 4 h incubation with iFluor 594-labeled micelles (Ex 563 nm, Em 604 nm). (**B**) iFluor 594 fluorescence intensity of micelles in 4T1 cells analyzed by flow cytometry

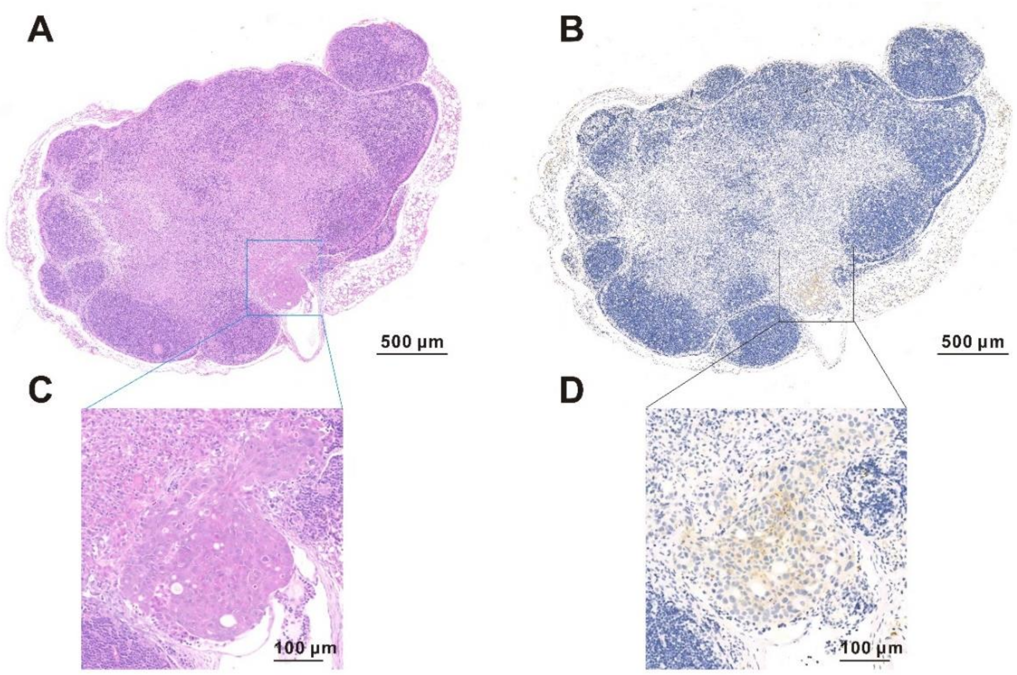

Fig. S12 Hematoxylin-eosin (**A**) and corresponding anti-HER2 immunohistochemical (**B**) staining of a representative metastatic mesenteric sentinel LN. The metastasis region in the LN was enlarged in **C** and **D**, respectively.

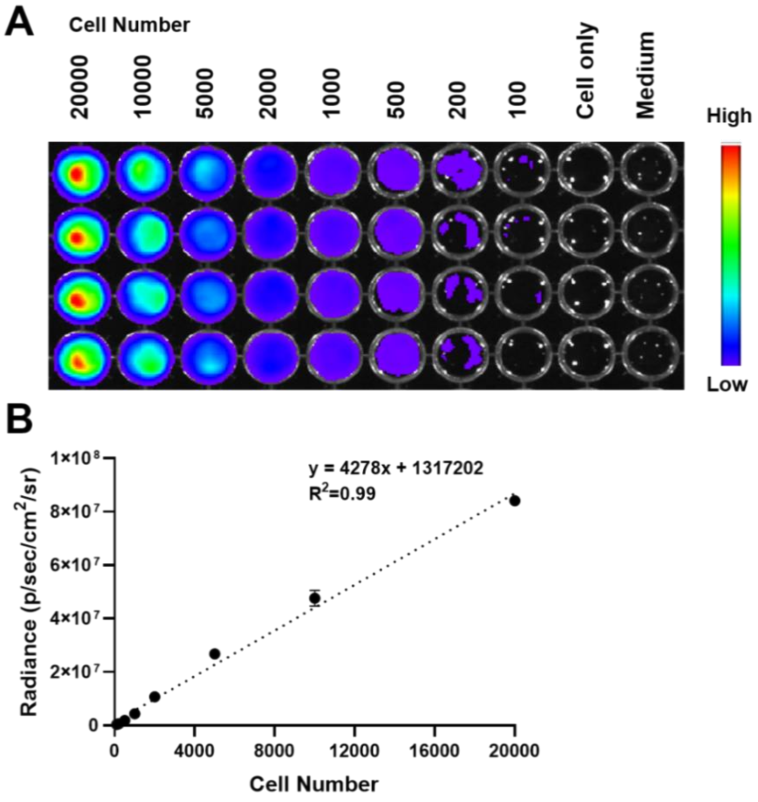

Fig. S13 Sensitive detection of the HT-29-luc cells using bioluminescence imaging. (**A**) Bioluminescence images of various cell number of tumor cells from 100-20000 cells. (**B**) Linear correlation between the bioluminescence signal intensity and tumor cell numbers. Data are presented as mean ± s.d. (n = 4)

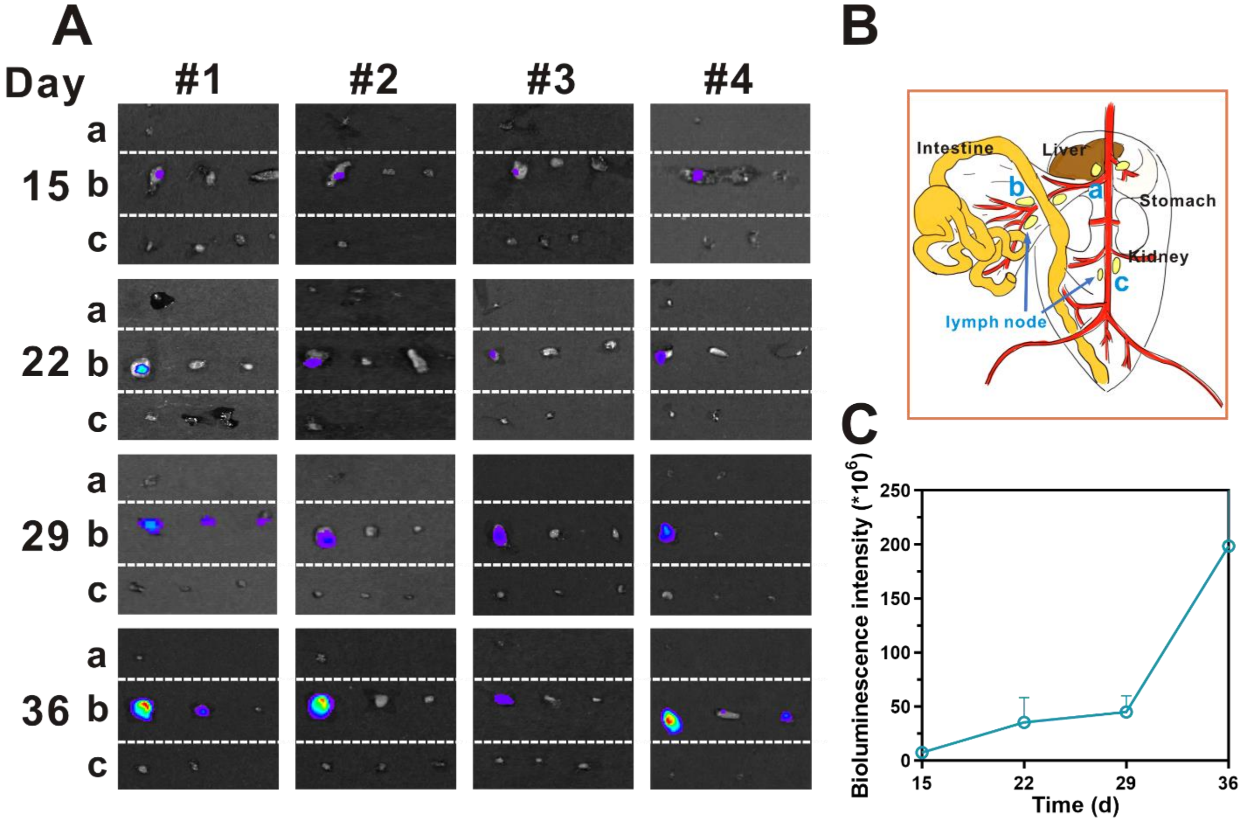

Fig. S14 LN metastasis profile of the orthotopic HT-29-luc colorectal tumor model. (**A**) LN metastasis was examined using bioluminescence imaging. 4 mice were included on each detection day (15, 22, 29, and 36 d). LNs at three anatomical positions (a. Pancreaticoduodenal LN; b. Mesenteric LN; c. Lumbar LN) were examined. (**B**) Schematic illustration of anatomical positions of the LNs. (**C**) Quantified bioluminescence intensity of the mesenteric sentinel LN. Data are presented as mean ± s.d. (n = 4)

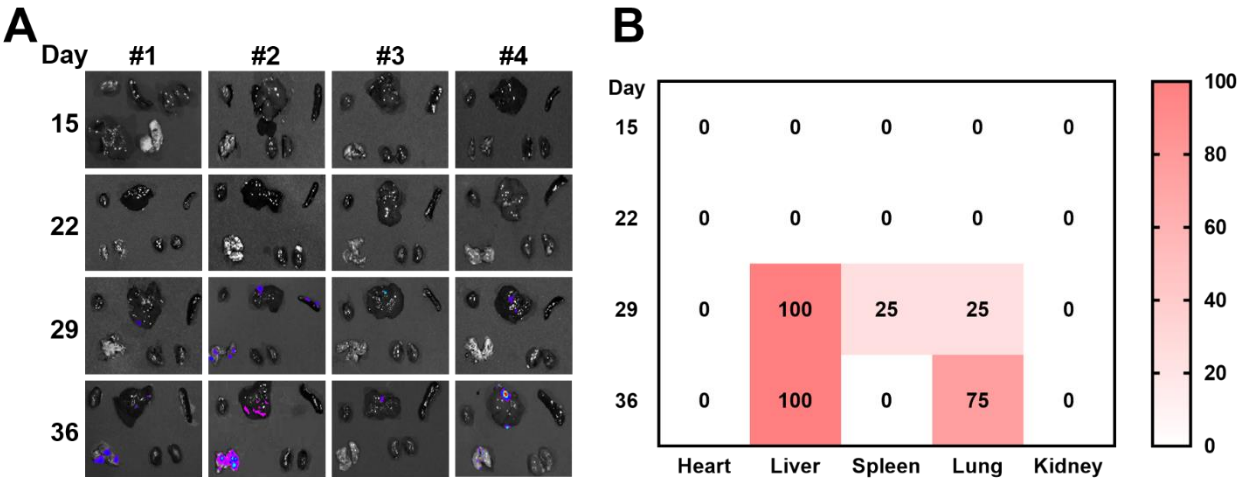

Fig. S15 Metastasis pattern in major organs of the orthotopic HT-29-luc colorectal tumor model. (**A**) The vital organs metastases were detected using the bioluminescence imaging from 15~36 days. Four mice were included on each detection day (15, 22, 29, and 36 d). (**B**) The time-dependent metastasis frequencies in the organs were summarized in the heat map

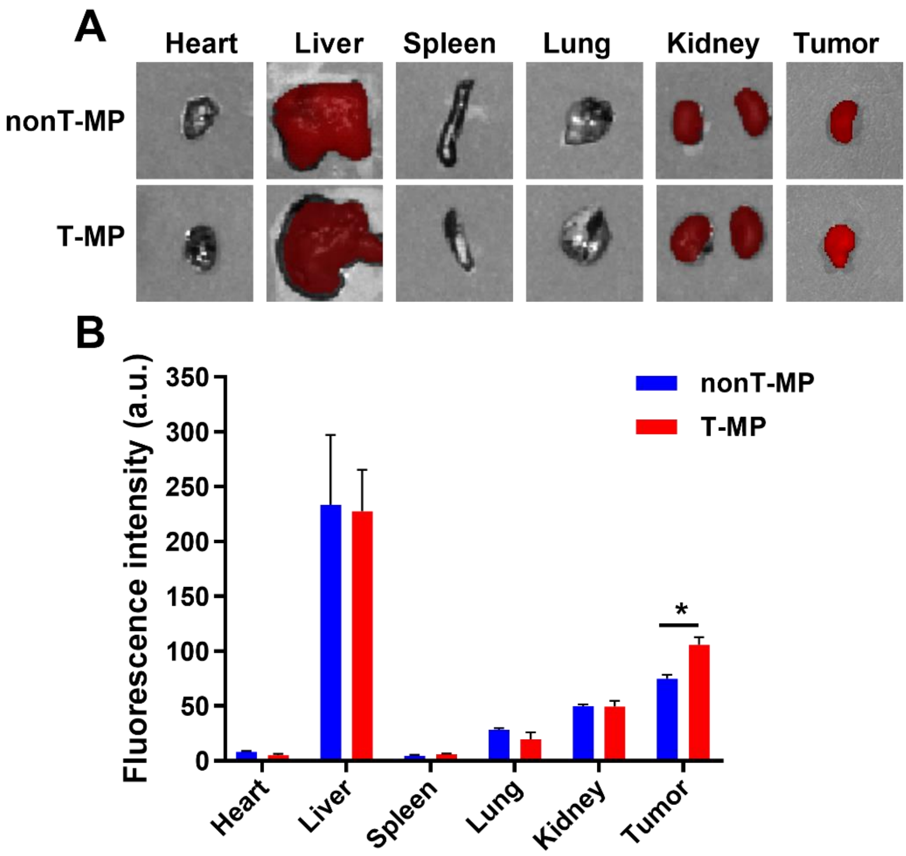

Fig. S16 Biodistribution of the Cy5.5 labeled micelles in major organs 8 h after i.v. injection. (**A**) Photos of the organs under IVIS Spectrum CT imaging system. (**B**) Quantified Cy5.5 fluorescence. Data are expressed as mean ± s.d. (n=3). *p < 0.05

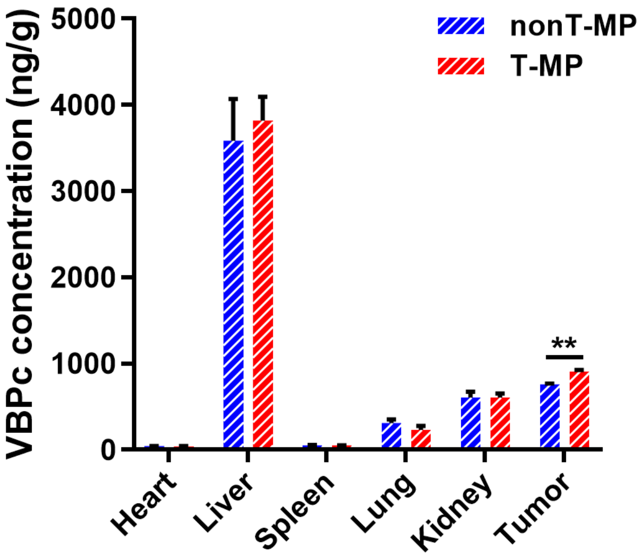

Fig. S17 Biodistribution of VBPc in major organs 8 h after i.v. injection. Data are expressed as mean ± s.d. (n=5). **p < 0.05

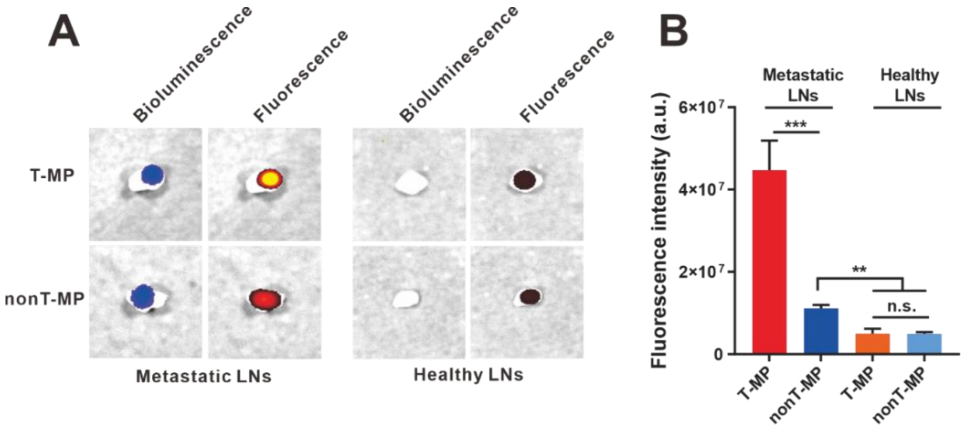

Fig. S18 Accumulation of micelles in metastatic LNs in the absence of HT-29-luc orthotopic tumors. (**A**) The orthotopic tumors were previously resected before the micelle injection. Representative ex vivo bioluminescence and fluorescence imaging of the metastatic LNs 8 h after i.v. injection of Cy5.5-labeled nonT-MP and T-MP. Their distribution in healthy LNs were also detected. (**B**) Quantified fluorescence intensity in the LNs in panel A. Data are presented as mean ± s.d. (n = 3)

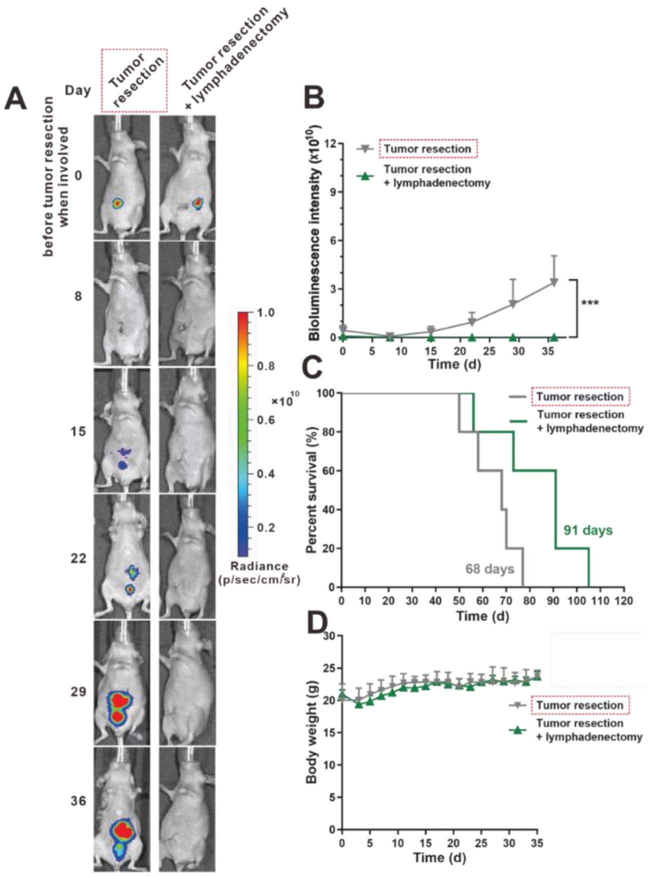

Fig. S19 The therapeutic outcome of tumor resection combined with lymphadenectomy. Tumor section alone indicated with dotted red line was used as control and also displayed in Figure 5 in the main text. (**A**) Representative in vivo bioluminescence imaging of the mice before tumor resection (day 0) and after tumor resection and photothermal therapy over the time. (**B**) Quantified bioluminescence intensity in panel A. (**C**) Survival curves of the mice. The median survivals of the mice were noted. (**D**) Mice body weight. Data are presented as mean± s.d. (n = 5).

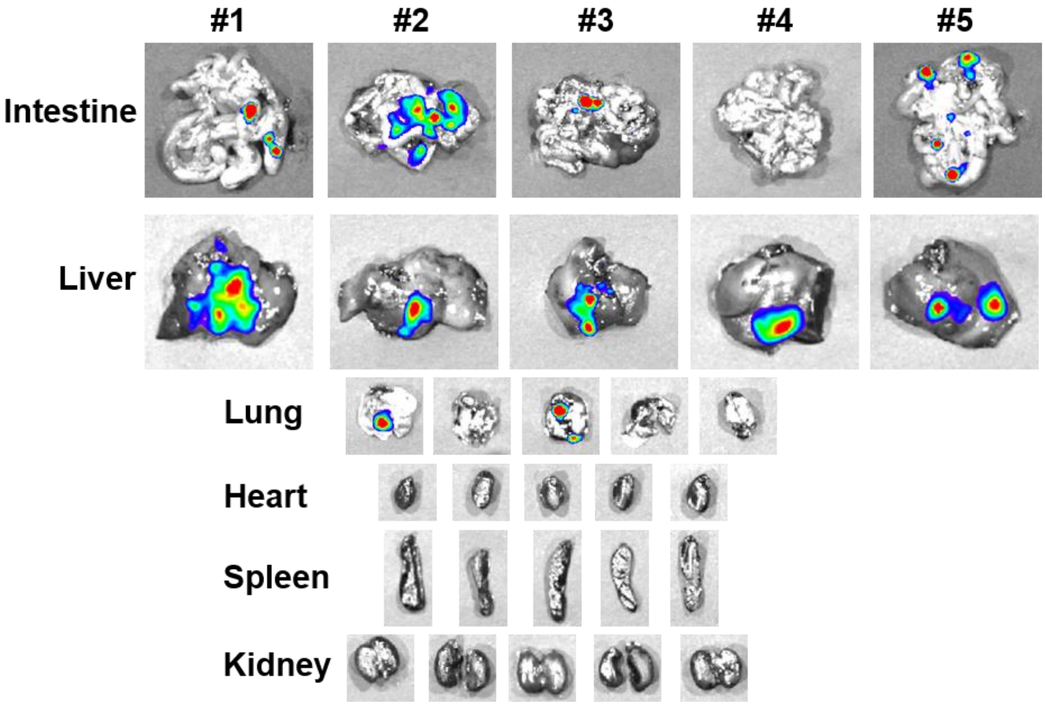

Fig. S20 When the mice of T-MP + Laser group died, the main organs (intestine, liver, lung, heart, spleen, and kidney) were excised for ex vivo bioluminescence imaging to examine the metastasis. The death times were day 75, 91, 96, 112, and 112 for mouse #1 to #5, respectively.

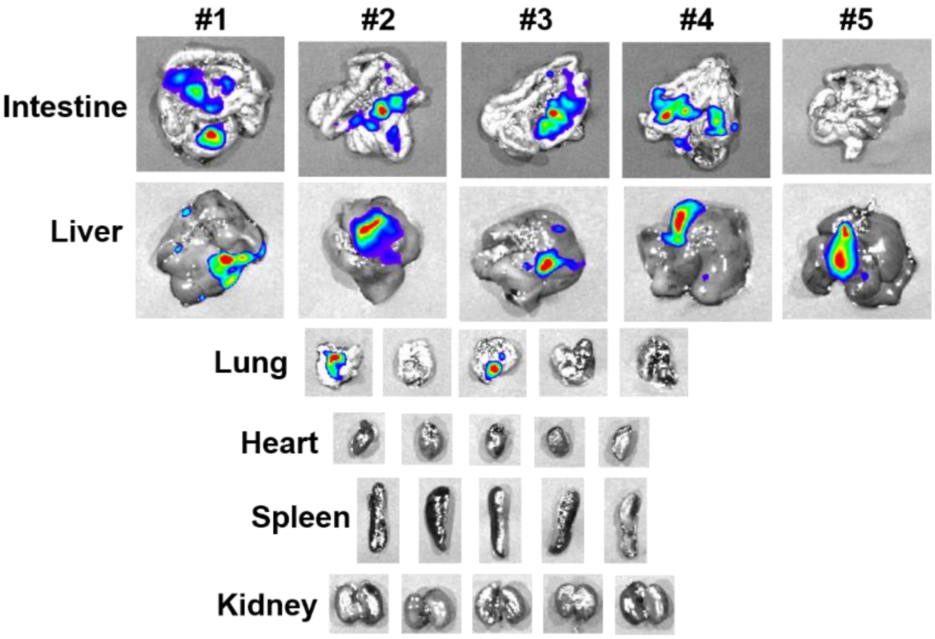

Fig. S21 When the mice of nonT-MP + Laser group died, the main organs (intestine, liver, lung, heart, spleen, and kidney) were excised for ex vivo bioluminescence imaging to examine the metastasis. The death times were day 57, 71, 75, 77, and 88 for mouse #1 to #5, respectively.

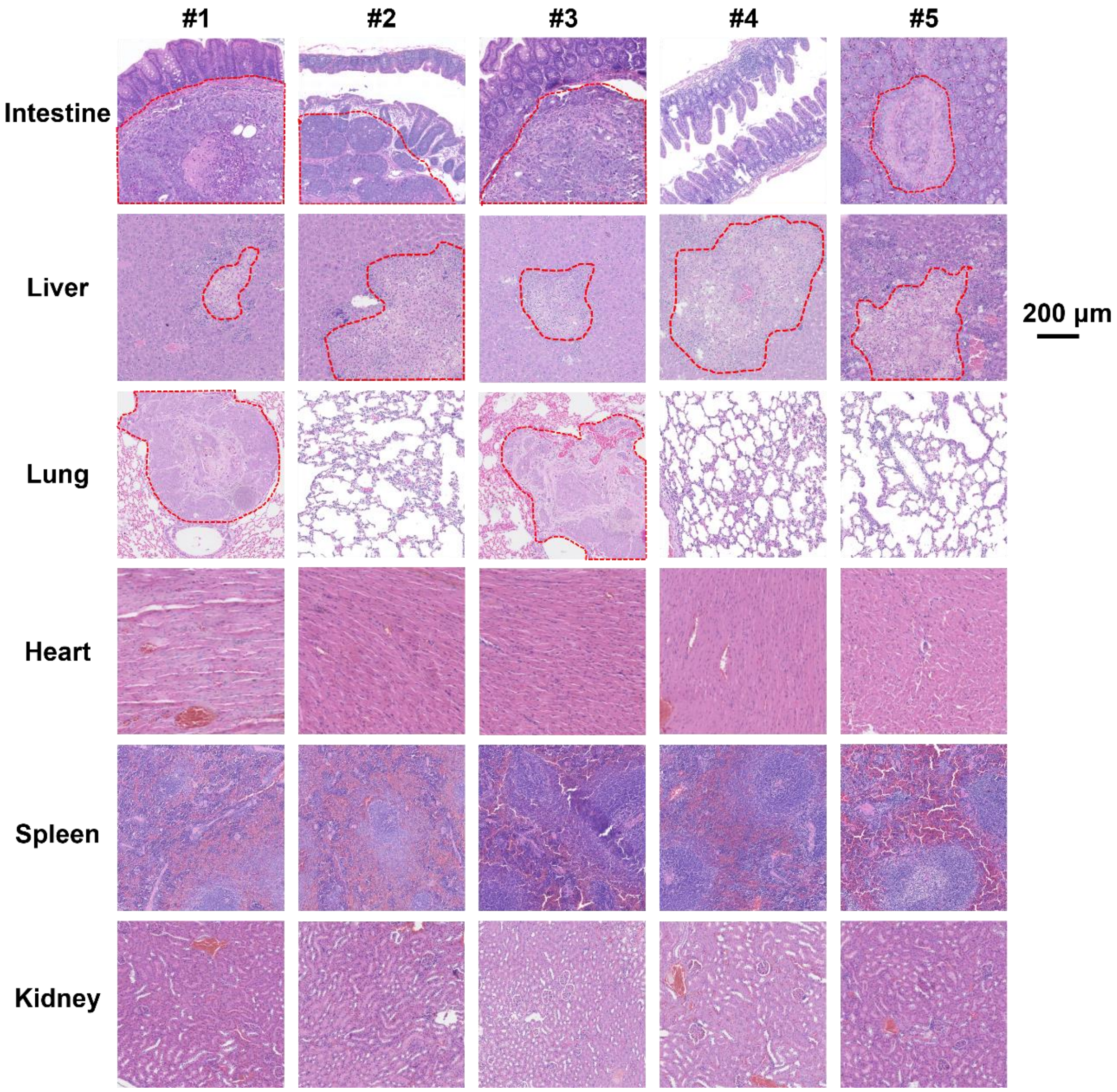

Fig. S22 When the mice of T-MP + Laser group died of the disease, the main organs (intestine, liver, lung, heart, spleen, and kidney) were excised and processed for paraffin sections and H&E staining to identify the metastasis. The red dotted line indicates the metastasis. The death times were day 75, 91, 96, 112, and 112 for mouse #1 to #5, respectively. Bar was 200 μm for all figures.

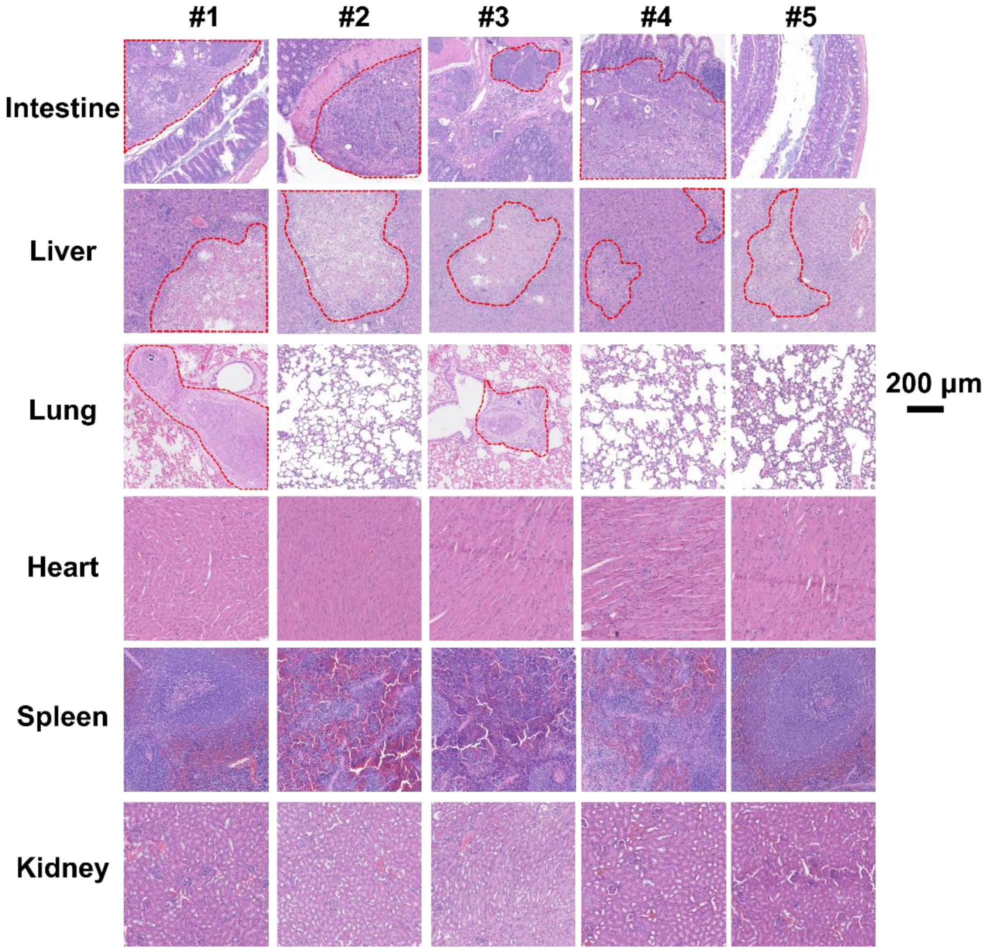

Fig. S23 When the mice of nonT-MP + Laser group died of the disease, the main organs (intestine, liver, lung, heart, spleen, and kidney) were excised and processed for paraffin sections and H&E staining to identify the metastasis. The red dotted line indicates the metastasis. The death times were day 57, 71, 75, 77, and 88 for mouse #1 to #5, respectively. Bar was 200 μm for all figures.

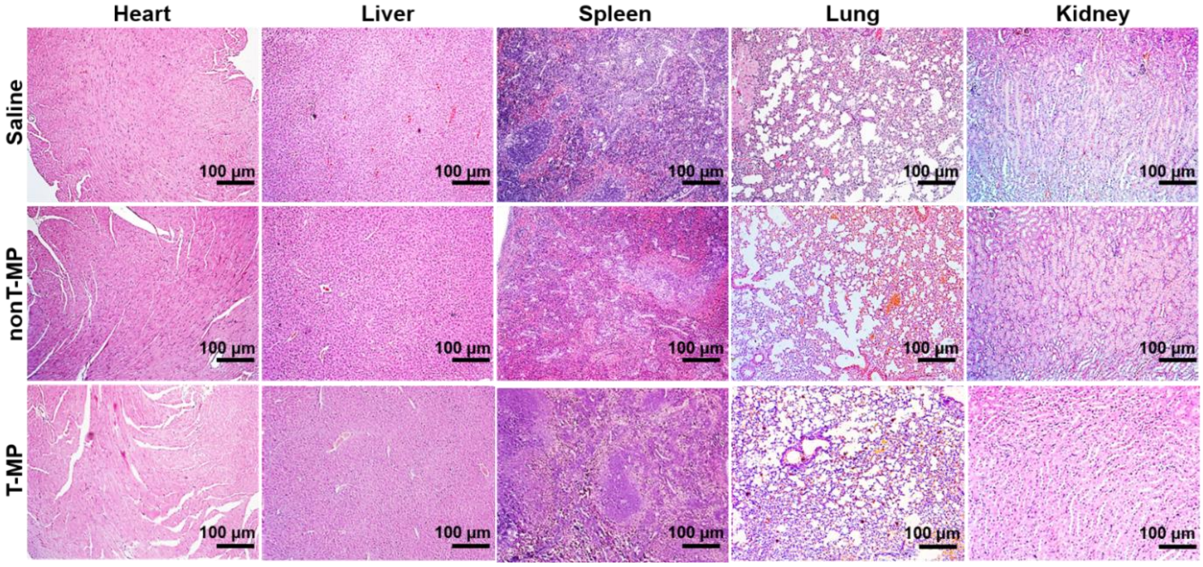

Fig. S24 24 h after micelle i.v. injection, major organs including heart, liver, spleen, lung, and kidney were excised and processed for H&E staining and pathological examination

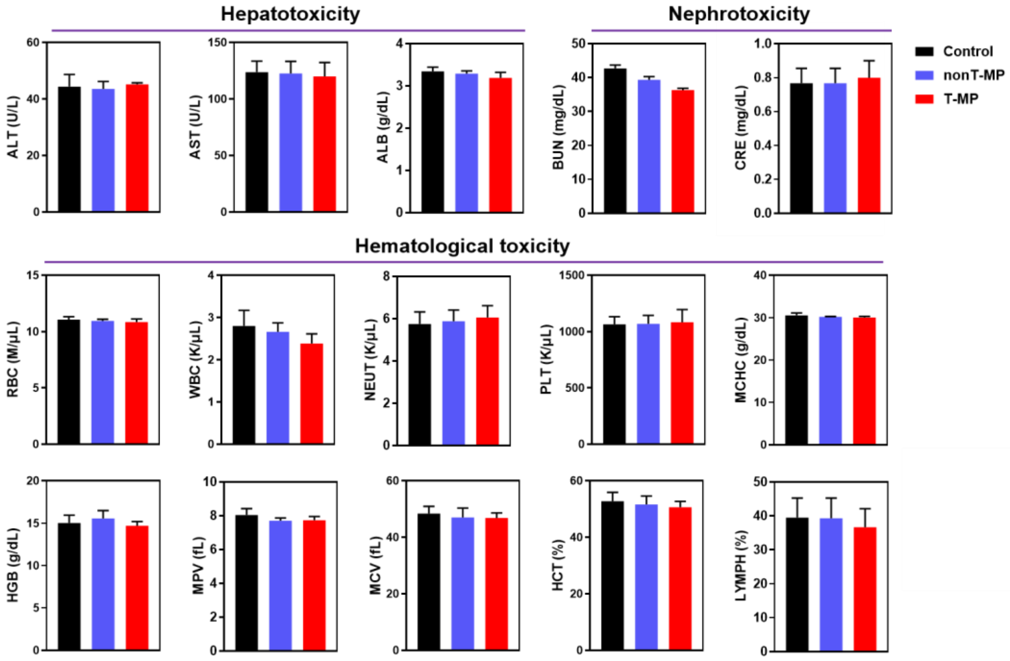

Fig. S25 Toxicity of the micelles in healthy female Balb/c mice. The micelles were i.v. injected to the mice. After 24 h, blood samples were collected from the mouse orbital for serum biochemistry and complete blood panel analysis. ALT, alanine aminotransferase. AST, aspartate aminotransferase. ALB, albumin. BUN, blood urea nitrogen. CRE, creatinine. RBC, red blood cells. WBC, white blood cells. NEUT, neutrophils. PLT, platelets. MCHC, mean corpuscular hemoglobin concentration. HGB, hemoglobin. MPV, mean platelet volume. MCV, mean corpuscular volume. HCT, hematocrit. LYMPH, lymphocytes. Data are presented as mean ± s.d. (n = 3).

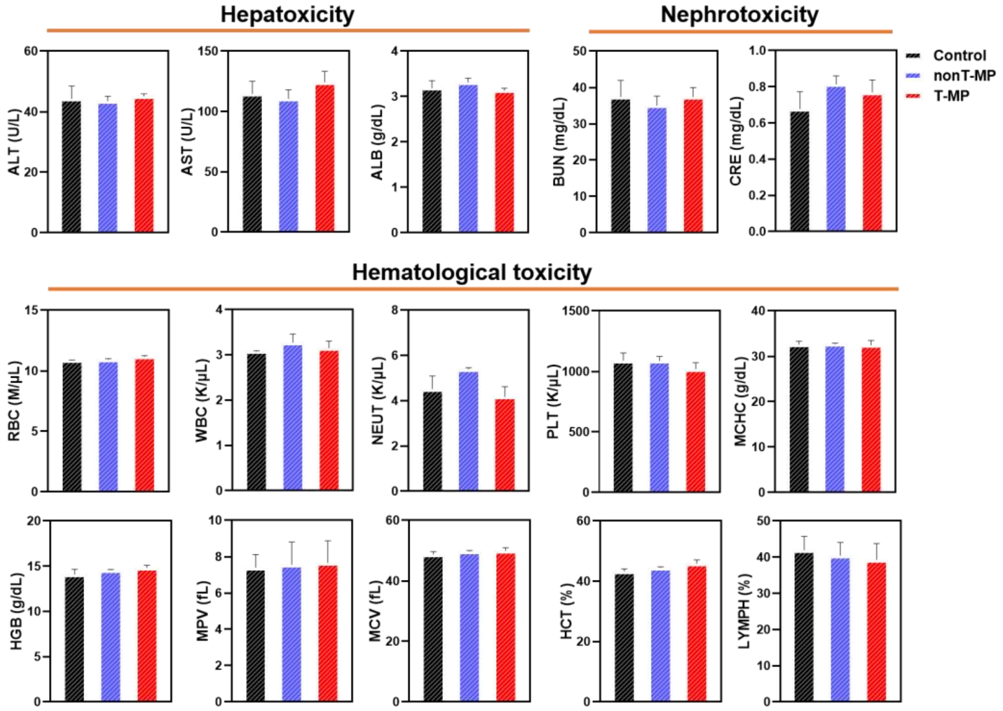

Fig. S26 After 96 h (4 days), blood samples were collected from the mouse orbital for serum biochemistry and complete blood panel analysis. Data are presented as mean ± s.d. (n = 3)

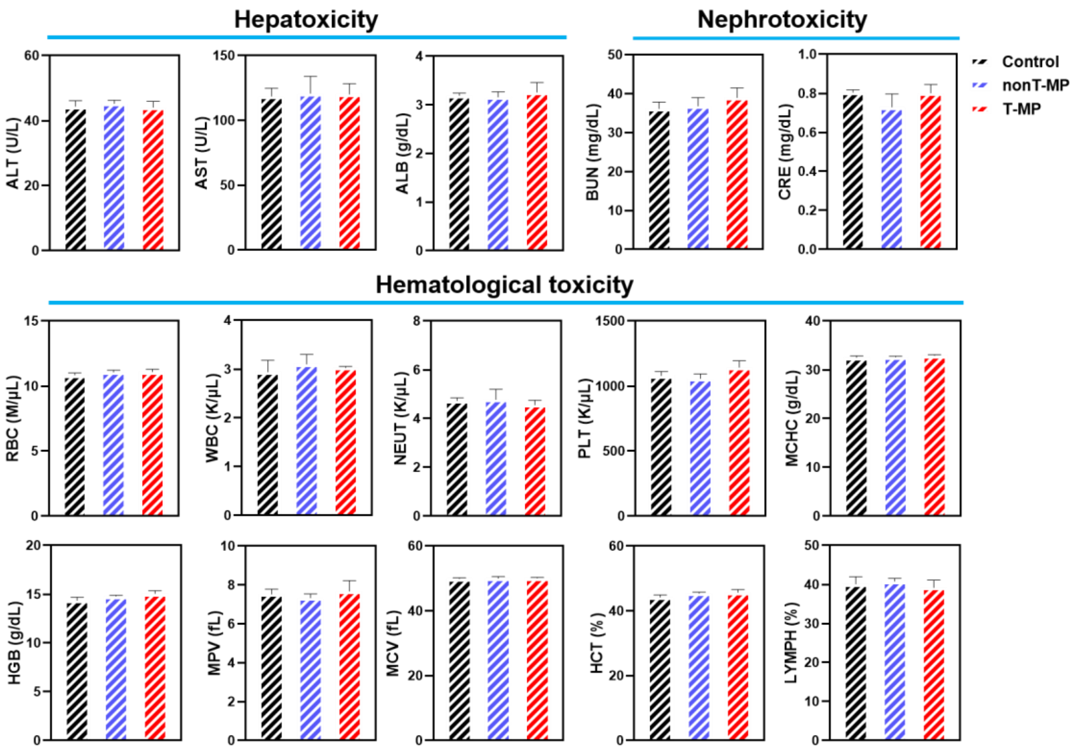

Fig. S27 After 168 h (7 days), blood samples were collected from the mouse orbital for serum biochemistry and complete blood panel analysis. Data are presented as mean ± s.d. (n = 3)

Table S1 Increase in life span (*ILS*) analysis of the mice after various treatments

		Median survival (day)	*ILS* (day) [a]	% *ILS* [b]
Tumor resection	Untreated	45	-	-
	Tumor resection	68	23	51.1
	nonT-MP	61	16	35.6
	T-MP	61	16	35.6
	nonT-MP + Laser	75	30	66.7
	T-MP + Laser	96	51	113.3

[a] *ILS*: Increase in Life Span (*T* - *C*), where *T* and *C* are the mean survival time of treated mice and control mice from the untreated group, respectively.

[b] $\%ILS = (T/C-1)\times 100\%$
